# *Sinorhizobium fredii* Strains HH103 and NGR234 Form Nitrogen Fixing Nodules With Diverse Wild Soybeans (*Glycine soja*) From Central China but Are Ineffective on Northern China Accessions

**DOI:** 10.3389/fmicb.2018.02843

**Published:** 2018-11-21

**Authors:** Francisco Temprano-Vera, Dulce Nombre Rodríguez-Navarro, Sebastian Acosta-Jurado, Xavier Perret, Romain K. Fossou, Pilar Navarro-Gómez, Tao Zhen, Deshui Yu, Qi An, Ana Maria Buendía-Clavería, Javier Moreno, Francisco Javier López-Baena, Jose Enrique Ruiz-Sainz, Jose Maria Vinardell

**Affiliations:** ^1^IFAPA, Centro Las Torres-Tomejil, Seville, Spain; ^2^Departamento de Microbiología, Facultad de Biología, Universidad de Sevilla, Avenida Reina Mercedes, Seville, Spain; ^3^Department of Botany and Plant Biology, University of Geneva, Geneva, Switzerland; ^4^Institute of Microbiology, Heilongjiang Academy of Sciences, Harbin, China; ^5^Departamento de Biología Celular, Facultad de Biología, Universidad de Sevilla, Seville, Spain

**Keywords:** *Sinorhizobium*, *Bradyrhizobium*, *Glycine max*, *Glycine soja*, rhizobia-legume symbiosis

## Abstract

*Sinorhizobium fredii* indigenous populations are prevalent in provinces of Central China whereas *Bradyrhizobium* species (*Bradyrhizobium japonicum*, *B. diazoefficiens*, *B. elkanii*, and others) are more abundant in northern and southern provinces. The symbiotic properties of different soybean rhizobia have been investigated with 40 different wild soybean (*Glycine soja*) accessions from China, Japan, Russia, and South Korea. Bradyrhizobial strains nodulated all the wild soybeans tested, albeit efficiency of nitrogen fixation varied considerably among accessions. The symbiotic capacity of *S. fredii* HH103 with wild soybeans from Central China was clearly better than with the accessions found elsewhere. *S. fredii* NGR234, the rhizobial strain showing the broadest host range ever described, also formed nitrogen-fixing nodules with different *G. soja* accessions from Central China. To our knowledge, this is the first report describing an effective symbiosis between *S. fredii* NGR234 and *G. soja*. Mobilization of the *S. fredii* HH103 symbiotic plasmid to a NGR234 pSym-cured derivative (strain NGR234C) yielded transconjugants that formed ineffective nodules with *G. max* cv. Williams 82 and *G. soja* accession CH4. By contrast, transfer of the symbiotic plasmid pNGR234a to a pSym^-^cured derivative of *S. fredii* USDA193 generated transconjugants that effectively nodulated *G. soja* accession CH4 but failed to nodulate with *G. max* cv. Williams 82. These results indicate that intra-specific transference of the *S. fredii* symbiotic plasmids generates new strains with unpredictable symbiotic properties, probably due to the occurrence of new combinations of symbiotic signals.

## Introduction

Rhizobia are α- and β-proteobacteria able to establish nitrogen-fixing symbioses with legumes (Sprent et al., 2017). The enormous ecological and economic importance of legume crops justifies the many extensive studies carried out these past four decades on rhizobia-legume symbioses. Different species belonging to the *Sinorhizobium* (=*Ensifer*) and *Bradyrhizobium* genera induce the formation of nitrogen-fixing nodules on soybean roots (Margaret et al., 2011; Yang et al., 2018). *Glycine max* [L.] Merr. (soybean) is the most important pulse legume in the world with its seeds as essential sources of proteins and oils. However, soybean is not only an important source of proteins and oils, but also a rich source of nutraceuticals compounds including bioflavonoids, lecithins, oligosaccharides, phytosterols, saponins, and tocopherols (Rosell et al., 2004; Hamilton-Reeves et al., 2007). *Glycine soja* (Siebold and Zucc.) is the wild ancestor of the domesticated soybean, *Glycine max* (recently reviewed by Kofsky et al., 2018). *G. soja* is native to East Asia and can be found in a broad geographic range that encompasses from East Russia to South China. Although wild and domesticated soybeans differ in many characteristics, the fact that they have the same number of chromosomes (2*n* = 40) and are cross-compatible make wild soybean very attractive as a potential genetic source of interesting traits that can have been partially or totally lost during domestication and improvement of *G. max* (Kofsky et al., 2018).

Soybeans are cropped on an estimated 6% of the world’s arable land and, in the last 40 years, areas under soybean production increased more than any other major crop (Hartman et al., 2011). Such continuous increase in soybean production is to match a growing demand for soybean-based meals and oils. Total world soybean production has increased from 17 million metric tons (MMT) in 1960 to 320 MMT in 2015, according to the USDA data (Westcott and Hansen, 2016). Soybean production is still expected to raise with improved yields and development of additional production areas. The increase in soybean cropping is concomitant with a burgeoning inoculant industry. In fact, the development of effective rhizobial inoculants for this crop has been the key to its spread into non-native regions of the world (Alves et al., 2003).

**FIGURE 1:**
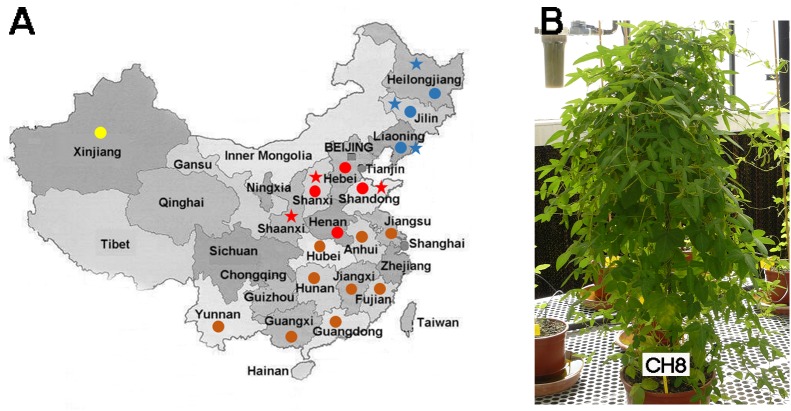
**(A)** Biogeography of *Glycine soja* cultivars analyzed in this work (stars) and soybean-nodulating rhizobia (circles) studied by Tian et al. (2012). Blue and red stars indicate Northern and Central Chinese provinces, respectively. Blue, red, brown, and yellow circles indicate Northern, Central, Southern, and Western Chinese provinces respectively. According to Tian et al. (2012), *Sinorhizobium* species are predominant in Central China whereas *Bradyrhizobium* species predominate in Northern and Southern China. In Xinjiang (Western China), the proportions of *Sinorhizobium* and *Bradyrhizobium* species are similar. **(B)** A wild *G. soja* cultivar (CH8, from Heilongjiang, Northern China) grown in greenhouse conditions in Seville, Spain.

China is the center of origin and diversification of soybean plants, where it has been cultivated for more than 5,000 years (Mammadov et al., 2018; Yang et al., 2018). According to ancient texts and mass spectrometry analyses of carbonized soybean seeds, the origin of soybean could be delimited from the North-eastern Hebei province to South-eastern areas of Northeast China. China should also be the center of origin of soybean rhizobia and the place where both symbionts coevolved. Apparently, the presence of root nodules was documented by Chinese writers from the very beginning of soybean cultivation, as illustrated by the ancient Chinese character “Shu” (soybean) that includes two dots below a horizontal stroke, which symbolizes root nodules belowground (Qiu and Chang, 2010). Slow-growing rhizobia isolated from either soybean or wild soybean nodules collected in Chinese soils belonged to diverse bradyrhizobia species including those currently classified as *Bradyrhizobium diazoefficiens*, *B. elkanii*, *B. japonicum*, *B. liaoningense*, and *B. yuanmingense*, while fast-growing soybean rhizobia belong to *Sinorhizobium fredii*, *S. sojae* or *Sinorhizobium* spp. (Tian et al., 2012; Zhao et al., 2014). The distribution of slow- versus fast-growing soybean rhizobia in Chinese provinces varies along a North–South axis (Tian et al., 2012), with fast-growers absent in Northern acidic soils (Heilongjiang, Jilin, and Liaoning provinces) that are mainly colonized by *Bradyrhizobium* species (Tian et al., 2012). Similarly, nodules of soybean plants grown in the acidic soils of Hubei, Anhui, and Jiangsu in Central China and all those provinces situated in the South mostly contain *Bradyrhizobium* strains (Figure 1) with few occurrences of *S. fredii* isolates (Tian et al., 2012). By contrast, the alkaline soil from Central China defined by the Huang-Huai-Hai rivers (Hebei, Shanxi, Henan, and Shandong provinces) mainly contains fast-growing soybean rhizobia, with *S. fredii* being the most abundant, as well as occasionally a few strains of *B. elkanii*, *B. liaoningense*, *B. yuanmingense*, and *Bradyrhizobium* spp. (Tian et al., 2012). Additional studies further highlighted the predominance of slow-growers in acidic soils while fast-growers dominated rhizobia populations in alkaline soils (Yang et al., 2018; Zhang et al., 2018). Thus, soil pH appears to be a decisive criterion in determining whether fast- or slow-growing soybean rhizobia communities will populate fields, including soils from alkaline spots of otherwise acidic soils of Honghu county (Hubei province) where *S. fredii* populations were found to be predominant (Camacho et al., 2002). In fact, sinorhizobial strains are often associated with legumes that are native to alkaline soils, not only in China but also in other countries such as India (Sankhla et al., 2017; Rathi et al., 2018).

The genetic differentiation and diversity from *Glycine soja* Sieb. & Zucc. to *G. max* [L.] Merr. has been studied at the DNA sequence level (Li et al., 2010, 2014; Zhou et al., 2015) and with proteomic analyses (Natarajan et al., 2007; Xu et al., 2007). Most of the reported results indicated that genetic diversity in *G. soja* was higher than in *G. max* (Qiu and Chang, 2010). The comparison of the genomes of seven *G. soja* accessions showed lineage-specific genes with variations in copy numbers and large-effect mutations, some of which may have contributed to the selection of important agronomic traits such as biotic resistance, composition of seeds, times of flowering and maturity, organ size and final biomass (Li et al., 2014).

Natural phenotypic changes affecting plant development, flowering time, seed size and color or dormancy have taken place during soybean domestication processes (Zhou et al., 2015; Mammadov et al., 2018). Wild soybeans (*G. soja*) can be a source of many elite traits, such as tolerance to salt (Luo et al., 2005), cold and dehydration stresses (Chen et al., 2006), or high lutein content (Kanamaru et al., 2006). Hence, wild soybeans can be used in breeding programs to obtain fertile hybrids of *G. max* and *G. soja* crosses (Singh, 2007; Li et al., 2010). In such *G. soja*/*G. max* hybrids, various important characteristics such as seed size, plant height and/or tolerance to waterlogging can be selected for, with some intermediate phenotypes occurring as well (Qiu and Chang, 2010).

In this work we compared the symbiotic capacity of 40 wild soybean accessions from Russia, South Korea, Japan, and China to associate with three different well-known soybean rhizobia strains: *B. diazoefficiens* USDA110^T^, *B. elkanii* USDA76^T^, and *S. fredii* HH103. Wild soybeans from North or Central China showed lower symbiotic efficiency with *S. fredii* HH103 than with bradyrhizobia strains USDA110^T^ and USDA76^T^. By contrast, in association with *G. soja* accessions from Central China, strain HH103 was equally or more efficient than USDA110^T^ and USDA76^T^ strains.

In addition, several *G. soja* accessions from Central China inoculated with *S. fredii* NGR234 were found to form nitrogen-fixing nodules. Out of 30 rhizobia isolated from nodules of *Lablab purpureus* collected in a slightly alkaline soil (pH 8.5) of Papua New Guinea, NGR234 was the only fast-growing strain (soil pH 8.5) (Trinick, 1980). Because of its unsurpassed host-range (Pueppke and Broughton, 1999), NGR234 became a model to study the molecular basis of symbiotic promiscuity (Broughton et al., 2000; Perret et al., 2000), although this strain failed to nodulate all *G. max* or *G. soja* varieties tested so far (Pueppke and Broughton, 1999). Thus, to our knowledge, this is the first report of NGR234 forming proficient nitrogen-fixing nodules with *G. soja* accessions.

## Materials and Methods

### Bacterial Strains, Plasmids, Culture Conditions, and Media

Bacterial strains and plasmids used in this study are listed in Supplementary Table S1. *S. fredii* strains were cultivated at 28°C in/on TY (Beringer, 1974), RMS (Broughton et al., 1986), or YMA (Vincent, 1970) media. *B. diazoefficiens* USDA110^T^ and *B. elkanii* USDA76^T^ were grown in/on YMA medium. *Escherichia coli* strains were cultivated on LB medium (Sambrook and Russell, 2001) at 37°C. When necessary, TY or YMA media were supplemented with streptomycin (50 μg/ml), rifampicin (50 μg/mL), chloramphenicol (5 μg/ml), kanamycin (25 μg/ml), neomycin (50 μg/ml) tetracycline (10 μg/ml), or spectinomycin (50 μg/ml). The isoflavone genistein was dissolved in ethanol and used at a final concentration of 1 μg/ml (3.7 μM). Plasmids were transferred from *E. coli* to rhizobia by conjugation as described by Simon (1984). Plasmid pMUS262 was used to detect conjugational transfer of symbiotic plasmids as described by Vinardell et al. (1993).

### Construction of *Sinorhizobium fredii* Hybrids by Intra-Specific Transfer of *S. fredii* Symbiotic Plasmids

To provide a selectable marker in the transfer of plasmids by conjugation, the *S. fredii* NGR234 symbiotic plasmid p*Sf*NGR234a was tagged with transposon Tn*5*-Mob, which confers neomycin resistance (Nm^R^). Random Tn*5*-Mob mutagenesis was carried out by crossing *E. coli* strain S17-1 carrying the suicide plasmid pSUP5011 with NGR234. A Nm^R^ derivative of NGR234, called NGR234-M, was identified by its capacity to transfer the Nm^R^ marker via a triparental cross between NGR234-M (as donor), *E. coli* HB101 (pRK2013) (as helper) and *Agrobacterium tumefaciens* GMI9023 Cm^R^ (pMUS262) (as recipient). Plasmid pMUS262 carries the *tet* gene from pBR322 [coding for tetracycline efflux MFS transporter Tet(C) protein, WP_010891057] under the control of a *nodA* promoter (p*nodA*). This construct only confers resistance to tetracycline when recipient cells are grown in presence of a flavonoid inducer, such as genistein, that activates the NodD1 transcriptional regulator for p*nodA::tet* expression (Vinardell et al., 1993). Thus, only the symbiotic plasmid p*Sf*NGR234a that carries a functional copy of *nodD1* could change the Nm^R^ *A. tumefaciens* strain GMI9023 Cm^R^ (pMUS262) into a transconjugant resistant to tetracycline when it was grown in the presence of genistein (Vinardell et al., 1993). Then, the symbiotic plasmid of NGR234-M carrying a Tn*5*-Mob insertion, now called p*Sf*NGR234a::Tn*5*-Mob or pSymNGR234M, was mobilized into the pSym cured derivative strain called *S. fredii* USDA193C (Buendía-Clavería et al., 1989). Using another tri-parental mating, the symbiotic plasmid of *S. fredii* HH103 marked with Tn*5*-Mob (called p*Sf*HH103d::Tn*5*-Mob or pSymHH103M; Romero, 1993) was transferred to ANU265 (here after called NGR234C), a derivative strain of NGR234 cured of pNGR234a (Morrison et al., 1983).

### Plant Tests

Nodulation tests on *G. max* (L.) Merr. cultivar Williams 82 (American soybean cultivar) were carried out as previously described (Buendía-Clavería et al., 1989; Yang et al., 2001). Leonard jars were used to grow plants for 6 weeks in a plant-growth chamber with a 16 h photoperiod at 25°C in the light and 18°C in the dark. *G. max* [L] and *G. soja* Sieb. & Zucc. (wild soybean) accessions from Japan, Korea, Russia, and China (Figure 1, Table 1, and Supplementary Table S2) were tested in a plant-growth chamber or in a greenhouse using Leonard jars. Because many *G. soja* accessions have very hard seed coats, treatments with sulfuric acid for 20 min followed by three washing steps with sterilized water were carried out. After this scarification treatment, seeds were surface sterilized with sodium hypochlorite 5% (w/v) for 5 min followed by six washes with sterilized water. Surface sterilized seeds were germinated on 1% (w/v) agar-water at 28°C for 2–3 days. Three or four germinated seeds were transferred to each sterilized Leonard jar consisting of a lower vessel with 500 ml of a pH 7 N-free half-strength nutrient solution (Rigaud and Puppo, 1975) supplemented with trace elements (Turner and Gibson, 1980) and an upper vessel with 300 ml of a mixture of moistened 2/1 (v/v) vermiculite-perlite (Camacho et al., 2002). Each seed was inoculated with approximately 10^8^ bacteria. Jars were placed in a greenhouse under natural light supplemented with light lamps to reach 14 h daylight with a daily minimum-maximum temperature of 18/32°C or in a plant-growth chamber with a photoperiod of 16 h light/day and a temperature range of 18–25°C (night–day). A week after emergence, seedlings were thinned to two per jar. Forty two or 60 days after inoculation, plant tops were dried at 80°C for 48 h and weighed. The relative efficiency index (REI), that measures the accumulation of N fixed in relation to the controls (Brockwell et al., 1966), was calculated for each inoculant/wild-soybean combination. REI was defined as (I-U/N-U) × 100, where (I), (U), and (N) correspond to dry-weight of shoots in each inoculation treatment (I) and in the corresponding uninoculated (U), and nitrogen-fertilized (N) controls. Each jar of the N-fertilized controls received 120 mg N, divided in three treatments of 30, 40, and 50 mg N that were applied at 15, 25, and 35 days of cultivation. Bacterial isolation from surface-sterilized nodules was carried out as previously described (Buendía-Clavería et al., 2003). These nodulation assays were carried out at IFAPA Research Center (Sevilla, Spain) and Harbin (China).

**Table 1:** Geographical origin and maturity group of *G. soja* accessions from China.

*G. soja* accession number in IFAPA collection	Other accession numbers^A,B^	Chinese Region or province	Locality	Maturity group	GPS coordinates	
					
					Latitude	Longitude
**Wild soybeans from Northern China**						
CH1	65549^A^	Heilongjiang	Sungari River	II	45° 49′	126° 41′
CH2	407288^A^	Jilin	Nan Wai Tse	II	43° 30′	124° 48′
CH3	549039^A^	Liaoning	Kuandian	III	40° 42′	125° 2′
CH6	ZYD00034^B^	Heilongjiang	Aihui	000	50° 22′	127° 53′
CH7	ZYD00093	Heilongjiang	Nenjiang	000	49° 17′	125° 20′
CH8	ZYD00173	Heilongjiang	Tongjiang	0	47° 67′	132° 50′
CH9	ZYD00527	Heilongjiang	Harbin	I	45° 75′	126° 63′
CH10	ZYD00728	Heilongjiang	Hailin	00	44° 57′	129° 35′
CH11	ZYD00792	Jilin	Qianguo	I	45° 17′	124° 83′
CH12	ZYD00893	Jilin	Jiutai	I	44° 15′	125° 83′
CH13	ZYD01060	Jilin	Wangqing	II	43° 32′	129° 75′
CH14	ZYD01164	Jilin	Lishu	II	43° 32′	124° 33′
CH15	ZYD01396	Jilin	Huinan	II	42° 68′	126° 03′
**Wild soybeans from Central China**						
CH4	597455^A^	Shanxi	Yuci	III	37° 40′	112° 51′
CH5	597458^A^	Shandong	Laixi	IV	36° 48′	120° 28′
CH16	ZYD03019	Shaanxi	Loufan	III	38° 05′	111° 78′
CH17	ZYD03101	Shaanxi	Wuxiang	III	36° 83′	112° 83′
CH18	ZYD03152	Shaanxi	Hejin	IV	35° 58′	110° 70′
CH19	ZYD03163	Shaanxi	Ruicheng	V	34° 71′	110° 68′
CH20	ZYD03183	Shaanxi	Linfen	IV	36° 08′	111° 50′
CH21	ZYD03721	Shanxi	Huanglong	IV	35° 60′	109° 86′
CH22	ZYD03735	Shanxi	Longxian	VI	34° 91′	106° 86′
CH23	ZYD03803	Shanxi	Yaoxian	IV	34° 91′	108° 98′
CH24	ZYD03830	Shanxi	Dali	V	34° 82′	109° 96′
CH25	ZYD03870	Shanxi	Tongguan	V	34° 56′	110° 25′


*^*A*^Plant introduction numbers given by the United States Department of Agriculture (USDA) collection.*

*^*B*^Accession numbers starting by ZYD are the plant introduction numbers given by the National Crop Genebank of China (NCGC).*

Additional nodulation tests on *G. soja* CH2 and CH4, and those on *Leucaena leucocephala*, *Tephrosia vogelii*, and *Vigna radiata* cv. King were carried out at the University of Geneva (Switzerland) using Magenta jars. Seeds were surface sterilized as previously described (Fumeaux et al., 2011) and incubated for 2 days, at 27°C and in the dark to germinate. Once germinated, seedlings were planted in Magenta jars (two seedlings per jar) containing sterile vermiculite (Lewin et al., 1990) and watered with nitrogen-free B&D nutritive solution (Broughton and Dilworth, 1971). After 2–4 days recovery, each seedling was inoculated with 2 × 10^8^ freshly grown bacteria. Plants were grown in controlled conditions with a 12 h photoperiod, a day temperature of 27°C and a night temperature of 20°C. To confirm the identity of bacteria found inside nodules, the 5′-end of the 23S rRNA genes of nodule bacteria was amplified using total genomic DNA of nodules as templates (Fossou et al., 2016) together with the 23S-For1 (5′-ACGAACTAGTGTCAAAAGGGC-3′) and ITS-Rev2 (5′-TGGTCCGCGTTCGCTCGCC-3′) primers. The resulting amplicons of NGR234 (317 bp) and HH103 (380 bp) were clearly discriminated after migration on a 1% agarose gel.

Experiments of competition for nodulation using pairs of co-inoculants among *S. fredii* HH103 Rif^R^, *B. diazoefficiens* USDA110^T^ or *B. elkanii* USDA76^T^, were carried out on *G. soja* accessions CH2, CH3, and CH4. In accession CH2, only the USDA110^T^/USDA76^T^ combination was used, since it does not nodulate with strain HH103 Rif^R^. Bacteria were grown to mid-log phase and *G. soja* plants were inoculated with 1 ml of a mixture of bacterial competitors containing 10^8^ (O.D. ∼ 0.1) bacteria at a 1:1 ratio. Plants were grown for 60 days under greenhouse conditions. Nodule occupancy was determined by the differential intrinsic antibiotic-resistance of the inoculants used: tetracycline (10 μg/ml) to positively differentiate bradyrhizobia (resistant) from HH103 Rif^R^ (sensitive) and streptomycin (50 μg/ml) to distinguish between USDA110^T^ (sensitive) and USDA76^T^ (resistant) bradyrhizobia strains. In addition, rifampicin (50 μg/ml) was also used to distinguish between USDA110^T^ (sensitive) and HH103 (resistant). These wild soybean accessions were also used in competition experiments between HH103 and its mutant derivatives affected in the T3SS HH103-1 *rhcJ*::Tn*5*::*lacZ* (=SVQ288) and HH103 Rif^R^ *ttsI*::Ω (=SVQ533). Nodule occupancy by mutants SVQ288 or SVQ533 was determined by plating bacterial nodule-isolates on TY media supplemented with neomycin (50 μg/ml) or spectinomycin (100 μg/ml), respectively.

### Microscopy Studies

Small fragments of nodules were fixed in 4% (v/v) glutaraldehyde prepared in 0.1 M cacodylate buffer, pH 7.2 for 1 h at 4°C. Samples were washed in 0.1 M cacodylate buffer, pH 7.2 for 12 h and post-fixed in 1% OsO_4_ for 1 h at 4°C. Then, samples were dehydrated in ethanol at progressively higher concentrations and embedded in Epon (epoxy embedding medium). Toluidine blue-stained semi-thin sections (0.5 μm thick) used as controls were viewed in a Leitz (Aristoplan) light microscope.

Thin sections (60-80 nm thick) were cut on a Reichert-Jung Ultracut E ultramicrotome, stained with uranyl acetate and lead citrate, and examined in a Libra 120 Plus transmission electron microscope (TEM) from Carl Zeiss (Germany) at an accelerating voltage of 80 kV.

## Results

### Symbiotic Efficacy of *B. diazoefficiens* USDA110^T^, *B. elkanii* USDA76^T^, and *S. fredii* HH103 Rif^R^ on Soybean Accessions From Russia, North Korea, Japan, and China

Twenty wild soybean (*G. soja*) accessions from Russia (R1 to R5), South Korea (K1 to K5), Japan (J1 to J5), and China (CH1 to CH5) from the USDA-Soybean Germplasm Collection were inoculated with three different soybean rhizobia strains: *B. diazoefficiens* USDA110T, *B. elkanii* USDA76^T^, and *S. fredii* HH103 Rif^R^. Two independent nodulation tests showed that USDA110^T^ and USDA76^T^ formed nitrogen fixing nodules with all accessions tested.

Some particular accessions, such as K3, K4, J2, R2, R5, CH1, CH2, CH3, and CH5 inoculated with USDA110^T^ produced REI values higher than 50%, and three accessions (K2, K5, and R3) gave REI values higher than 80% (Table 2 and Supplementary Table S3). The highest REI value in *G. soja* plants inoculated with USDA76^T^ was obtained with accession R4 (57.7%). REI values higher than 40% were found with Korean (K2 and K3), Russian (R3), Northern (CH1 and CH2) and Central China (CH3, CH4, and CH5) accessions (Table 2 and Supplementary Table S4). Wild-soybean accessions from Russia, Korea, Japan, and Northern China nodulated with HH103 Rif^R^ but REI values were lower than 5% (Table 2 and Supplementary Table S5). Accession CH2 (Jilin province, Northern China) failed to nodulate with HH103 Rif^R^. In contrast, one of the accessions tested from Central China (CH4, Shanxi province) gave a REI value of 54%, which was associated with healthy plant growth and absence of nitrogen deficiency symptoms (Supplementary Table S5).

**Table 2:** Relative Efficiency Indexes (REI) of *Glycine soja* accessions from Korea, Japan, Russia, and China when grown in combination with *B. diazoefficiens* USDA110^T^, *B. elkanii* USDA76^T^ or *S. fredii* HH103 Rif^R^.

Country (Province)	*G. soja* accession	REI of *G. soja* accessions inoculated with		
		
		USDA110^T^	USDA76^T^	HH103 Rif^R^
Korea	K1	26.0	15.1	0.2
	K2	98.3	43.8	1.4
	K3	51.9	46.5	1.0
	K4	59.8	28.4	3.9
	K5	86.6	23.7	0.5
Japan	J1	35.0	18.1	0.6
	J2	51.8	33.4	0.9
	J3	48.1	19.7	0.8
	J4	36.6	18.1	1.5
	J5	31.4	6.9	0.0
Russia	R1	32.1	38.0	2.1
	R2	51.0	18.3	2.9
	R3	96.7	53.0	0.8
	R4	38.9	57.7	2.3
	R5	55.1	41.9	2.7
North of China (Heilongjiang)	CH1	59.3	40.5	2.1
	CH6	89.9	19.2	4.7
	CH7	33.1	13.9	2.7
	CH8	31.7	18.4	2.7
	CH9	50.5	18.5	2.7
	CH10	29.9	26.9	9.2
North of China (Jilin)	CH2	59.9	47.7	0.0
	CH11	31.1	33.9	4.1
	CH12	48.2	16.2	0.2
	CH13	37.8	12.6	0.7
	CH14	30.2	8.5	0.2
	CH15	48.3	8.7	2.6
North of China (Liaoning)	CH3	55.1	44.3	4.4
East-Central coastal China (Shandong)	CH5	50.1	42.6	1.9
Central China (Shaanxi)	CH16	44.5	41.8	34.3
	CH17	37.9	17.5	56.3
	CH18	35.5	45.5	30.2
	CH19	29.6	46.3	44.1
	CH20	46.3	17.1	67.4
Central China (Shanxi)	CH4	31.7	48.8	54.0
	CH21	12.0	11.3	5.6
	CH22	23.7	10.8	2.1
	CH23	41.4	20.0	4.2
	CH24	33.8	37.4	43.5
	CH25	40.7	44.9	65.9


*The Relative Efficiency Index (REI) for each inoculant/soybean-accession combination was calculated by comparisons among Shoot Dry Weight of inoculated treatments (I), N-fertilized (N) and Untreated (U) controls. REI values were obtained by using the relation (I - U/N - U) × 100.*

Indigenous *S. fredii* populations are predominant in soils of Central China while *B. japonicum* and other slow-growing soybean-rhizobial strains are more abundant in soils of Northern and Southern China (Figure 1; Tian et al., 2012). To investigate whether the relative abundance of fast- and slow-growing populations of indigenous soybean rhizobia in particular areas correlates with symbiotic preferences of local *G. soja* accessions to nodulate with slow- or fast-growing soybean rhizobia, 10 accessions from Shanxi and Shaanxi provinces (Central China) and 10 wild soybeans from Heilongjiang and Jilin provinces (Northern China) were tested in combination with USDA110^T^, USDA76^T^, or HH103 Rif^R^. USDA110^T^ formed nitrogen-fixing nodules with the 20 wild soybeans tested. REI values close or equal to 50% were found with accessions CH9, CH12, and CH15, all from Northern China (Table 2 and Supplementary Table S6). One accession (CH6) from Heilongjiang province gave a remarkably high REI value of 89.9%. USDA76^T^ also nodulated all of the 20 accessions (Supplementary Table S7). None of the accessions from Northern or Central China gave REI values higher than 46.5%. Nine out of the 10 accessions from Northern China inoculated with HH103 Rif^R^ formed nodules, although REI values were below 9.5% (Table 2 and Supplementary Table S8). In contrast, all of the 10 accessions from Central China were nodulated by HH103 Rif^R^ and 5 of them gave REI values in the range of 43–67%.

### Competition Experiments Between *B. diazoefficiens* USDA110^T^, *B. elkanii* USDA76^T^ and *S. fredii* HH103 Rif^R^ to Nodulate Different Wild Soybean Accessions

HH103 Rif^R^ formed nitrogen-fixing nodules with *G. soja* accessions CH3 (REI 4.4%) and CH4 (REI 54%). Competition experiments for nodulation of accessions CH3 and CH4 were carried out between HH103 Rif^R^ and USDA110^T^ or USDA76^T^. Both bradyrhizobial strains outcompeted HH103 Rif^R^ to nodulate CH3 and CH4. HH103 Rif^r^ did not form nodules with accession CH3 when co-inoculated with both bradyrhizobial strains. On CH4, nodules occupied exclusively by HH103 Rif^R^ were less than 20% of the total number of nodules analyzed (Table 3). Similar experiments carried out between USDA110^T^ and USDA76^T^ showed that strain USDA110^T^ was always more competitive with accession CH2 and CH3 from Northern China, but less competitive with accession CH4 from Central China (Table 3).

**Table 3:** Nodule occupancy of *Glycine soja* accessions CH2, CH3, and CH4 inoculated with pairs of competitors formed among *B. diazoefficiens* (Bd) USDA110^T^, *B. elkanii* (Be) USDA76^T^ or *S. fredii* (Sf) HH103 Rif^R^.

*G. soja* accession	Pairs of inoculants (A/B)	TNN	% of nodule occupancy		
			
			A	B	A + B
CH2	BdUSDA110^T^/BeUSDA76^T^	191	46.6	23.0	30.4
CH3	BdUSDA110^T^/SfHH103 Rif^R^	186	93.5	0	6.5
	BeUSDA76^T^/SfHH103 Rif^R^	192	100	0	0
	BdUSDA110^T^/BeUSDA76^T^	189	70.9	9.5	19.6
CH4	BdUSDA110^T^/SfHH103 Rif^R^	181	78.5	12.7	8.8
	BeUSDA76^T^/SfHH103 Rif^R^	190	75.8	12.1	12.1
	BdUSDA110^T^/BeUSDA76^T^	192	27.6	68.2	4.2


*TNN, Total number of nodules tested in two independent experiments. In each experiment, 6 plants were inoculated per pair of competitors. Streptomycin (50 μg/ml) was used to distinguish between S. fredii HH103 Rif^*R*^ or B. diazoefficiens USDA110^*T*^ (sensitive) and B. elkanii USDA76^*T*^ (resistant). Rifampicin (50 μg/ml) and tetracycline (10 μg/ml) were used to distinguish between S. fredii HH103 Rif^*R*^ (rifampicin-resistant and tetracycline-sensitive) and B. diazoefficiens USDA110^*T*^ (rifampicin-sensitive and tetracycline-resistant).*

### *S. fredii* NGR234 Forms Nitrogen-Fixing Nodules With Wild Soybeans From Central China

**FIGURE 2:**
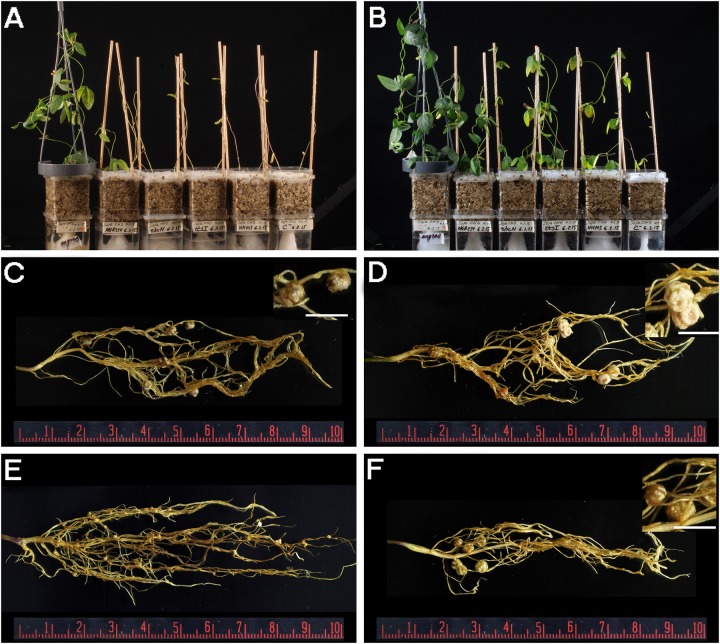
**(A,B)** Plant responses of *G. soja* accessions CH2 **(A)** and CH4 **(B)** inoculated with different *S. fredii* strains. From left to right, Magenta jars were treated as follows: N-fertilizer, NGR234, NGRΩ*rhcN*, NGRΩ*ttsI*, HH103 Rif^R^, and uninoculated control. **(C,D)** Nitrogen-fixing nodules induced by NGR234 on CH2 **(C)** and CH4 **(D)** roots. **(E)** Ineffective nodules induced by HH103 Rif^R^ on CH2 roots. **(F)** Nitrogen-fixing nodules induced by HH103 Rif^R^ on CH4 roots. White bars **(C,D,F)** correspond to 5 mm.

**FIGURE 3:**
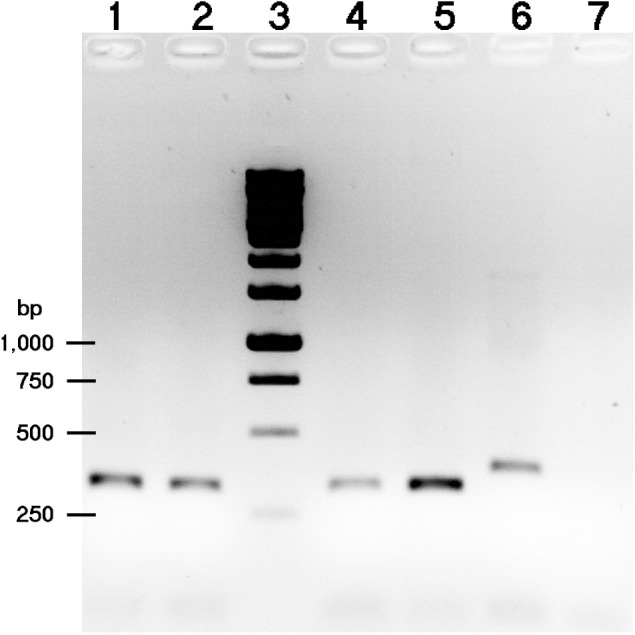
Separation of 23S-For1+ITS-Rev2 PCR amplified products on 1% agarose gel. The templates used were as follows: lanes 1, 2, and 4, gDNA from bacteria isolated from different nodules of *G. soja* CH2 inoculated with NGR234; lane 5, NGR234 gDNA; lane 6, HH103 Rif^R^ gDNA; lane 7, no DNA. Lane 3, DNA molecular weight marker (bands corresponding to 1,000, 750, 500, and 250-bp are indicated on the left).

Since the *G. soja* accession CH4 (Central China) formed proficient symbiosis with HH103 Rif^R^, we tested whether NGR234 was also capable of inducing the formation of nitrogen-fixing nodules on CH4 roots. Accession CH2 (Northern China), which was unable to nodulate with HH103, was also included in the nodulations tests. CH4 plants inoculated with NGR234 formed nitrogen-fixing nodules that effectively contributed to plant growth (Table 4 and Figure 2), whereas on CH2 accession NGR234 induced the formation of more nodules than on CH4 but plants were poorly developed. To our knowledge, this is the first report showing that *S. fredii* NGR234 can form proficient symbiosis with *G. soja* plants. Then, bacteria from nodules formed on CH2 and CH4 plants were isolated and identified. Isolates from well-developed nodules of CH2 and CH4 plants inoculated with NGR234 were resistant to rifampicin, a chromosomal antibiotic-resistance maker for this strain. To further confirm the presence of NGR234, a pair of PCR primers that specifically amplifies the 5′ end of the 23S rRNA gene was used. These primers can also be used to distinguish between NGR234 and HH103 strains, since the size of the predicted amplicons are of 317 and 380 bp, respectively. PCR using bacteria isolated from nodules formed on CH2 plants inoculated with NGR234 only amplified the DNA fragment corresponding to this strain (Figure 3).

**Table 4:** Symbiotic responses of *Glycine soja* accessions CH2, CH3, and CH4 to inoculation with diverse *S. fredii* HH103 and NGR234 mutants affected in the Type Three Secretion System (T3SS).

Treatments	Number of	Dry weight of	Shoot dry
	nodules	nodules (mg)	weight (mg)
***Glycine soja* CH2**			
HH103-1	0.0	0.0	15.7 ± 5.2b
SVQ288	7.1 ± 1.4a	13.0 ± 1.5a	49.1 ± 9.6b
SVQ533	7.0 ± 4.1a	16.6 ± 9.7a	96.8 ± 58.8a
Uninoculated	0.0	0.0	18.2 ± 9.0b
NGR234	16.2 ± 16.8	3.75 ± 4.6	105.6 ± 70.5a
NGRΩ*rhcN*	0.0	0.0	16.3 ± 13.4b
NGRΩ*ttsI*	0.0	0.0	23.3 ± 14.3b
Uninoculated	0.0	0.0	18.2 ± 9.0b
***Glycine soja* CH3**			
HH103-1	17.4 ± 6.4a	42.3 ± 11.1a	243.5 ± 61a
SVQ288	12.1 ± 6.3a	31.3 ± 6.1ab	213.5 ± 19ab
SVQ533	9.6 ± 7a	19.0 ± 8.8b	170.1 ± 55.6b
Uninoculated	0.0	0.0	14.5 ± 2.2c
NGR234	2.0 ± 3.3	4.9 ± 5.8	101.5 ± 88.8a
NGRΩ*rhcN*	0.0	0.0	27.8 ± 9b
NGRΩ*ttsI*	0.0	0.0	32.8 ± 14.1b
Uninoculated	0.0	0.0	14.5 ± 2.2b
***Glycine soja* CH4**			
HH103-1	26.1 ± 8.1a	59.4 ± 23.8a	858.0 ± 316.2a
SVQ288	12.6 ± 8.4b	52.9 ± 35.4a	762.8 ± 398.6a
SVQ533	14.9 ± 1.6b	60.8 ± 7a	774.5 ± 132.7a
Uninoculated	0.0	0.0	23.0 ± 5.8b
NGR234	6.6 ± 1.9a	19.0 ± 5.2a	269.8 ± 70.7a
NGRΩ*rhcN*	4.5 ± 3.6ab	13.6 ± 8.5a	262.9 ± 116a
NGRΩ*ttsI*	2.7 ± 2.3bc	3.8 ± 4.8b	61.7 ± 33.1b
Uninoculated	0.0	0.0	23.0 ± 5.8b


*Numbers refer to mean values (±standard deviations) per plant of three Leonard jars, each containing two plants. Statistical comparisons among inoculations are shown using ANOVA. For each parameter, data followed by the same letter are not significantly different at α = 5%.*

To investigate whether NGR234 was also able to effectively nodulate other wild soybean accessions from Central China, six accessions from Shanxi or Shaanxi provinces were inoculated with this strain. HH103 Rif^R^ was used as positive control in these experiments, which showed that NGR234 formed nitrogen-fixing nodules on all of the six accessions tested, albeit associations were clearly less proficient than those formed by HH103 Rif^R^ (Table 5).

**Table 5:** Symbiotic responses of some *Glycine soja* accessions from Central China with *S. fredii* HH103 and NGR234 strains.

Accessions	Number of nodules		Dry weight of nodules (mg)		Shoot dry weight (mg)		
			
	HH103	NGR234	HH103	NGR234	HH103	NGR234	Uninoculated
CH16	94.3 ± 13.2a	27.5 ± 13.5cde	110.3 ± 25.2abcd	63.5 ± 23.5bcde	1459 ± 337de	737 ± 66fg	118 ± 22gh
CH17	50.7 ± 3.4abcde	3.3 ± 1.9e	175.0 ± 16.4a	15.0 ± 4.9e	2340 ± 90ab	324 ± 12gh	79 ± 27h
CH19	86.7 ± 13.9ab	10.0 ± 0.6de	143.7 ± 19.7ab	55.7 ± 1.8cde	2040 ± 210abcd	1017 ± 112ef	124 ± 28gh
CH20	82.7 ± 25.1abc	88.7 ± 44.2ab	174.0 ± 57.4a	112.7 ± 51.2abc	2293 ± 408ab	1192 ± 561ef	122 ± 1gh
CH24	77.3 ± 30.8abc	59.3 ± 27.8abcde	136.0 ± 45.6abc	107.0 ± 28.4bcd	2177 ± 308abc	1552 ± 383cde	150 ± 19gh
CH25	47.3 ± 6.2abcde	35.3 ± 20.6bcde	194.3 ± 20.7a	60.0 ± 20.2bcde	2647 ± 421a	709 ± 287fgh	146 ± 18gh
CH4	62.0 ± 17.2abcd	13.3 ± 2.0de	167.3 ± 23.5a	23.7 ± 1.8de	1970 ± 257bcd	595 ± 47fgh	96 ± 1gh
LSD (*p* < 0.05)	58.6		86.8		646		


*Numbers are mean values (±standard error of the mean, n = 3) of three Leonard jars, each containing two plants. Determinations were carried out 60 days after inoculation. For each parameter, data followed by the same lower-case letter are not significantly different at α = 5%, as determined by protected Fisher’s LSD (Least Significant Difference).*

### Type Three Secretion Systems (T3SS) of *S. fredii* HH103 and NGR234 Determine Symbiotic Compatibility With Some *G. soja* Accessions

It has been established that secretion of nodulation outer proteins (Nop) through type three secretion systems (T3SS) modulates symbiotic compatibility of *S. fredii* strains with different *G. max* cultivars and other legume species (for a review see López-Baena et al., 2016). T3SS deliver effector proteins into the cytoplasm of animal and plant cells. In rhizobia, genes encoding structural components of symbiotically active T3SS and effector proteins secreted by these systems are controlled by a transcriptional regulator called TtsI (for a recent review see Staehelin and Krishnan, 2015). Amongst the many proteins assembled into a functional secretion machinery, RhcJ (first annotated as NolT in *S. fredii* strain USDA257) is embedded in the protein ring that spans the inner membrane of bacteria. By contrast, the nodulation outer protein (Nop) NopJ is one of the several effectors that together contribute to define the host range of strain NGR234 (Kambara et al., 2009). To test whether Nop-secretion also contributed to symbioses with wild soybean accessions from different regions of China, the nodulation properties of parent (HH103 and NGR234) and T3SS-deficient (SVQ288, a mutant of HH103 in RhcJ, and NGRΩ*rhcN*) strains were compared.

Neither a streptomycin-resistant derivative (*S. fredii* HH103-1) nor a rifampicin resistant mutant (*S. fredii* HH103 Rif^R^) of HH103 nodulated *G. soja* CH2, an accession from Northern China (Table 4 and Supplementary Table S5). Interestingly, mutants of *S. fredii* HH103 in *ttsI* (strain SVQ533) or *rhcJ* (strain SVQ288), both of which cannot secrete Nops, induced the formation of nodules (Table 4). These nodules, however, poorly contributed to plant growth. Conversely, *S. fredii* NGR234 that induced the formation of nitrogen-fixing nodules with *G. soja* CH2, completely lost its nodulation capacity when T3SS was inactivated as in mutants NGRΩ*rhcN* or NGRΩ*ttsI* (Table 4). Both *S. fredii* strains HH103-1 and NGR234 induced the formation of nodules with *G. soja* accession CH3 (Northern China), with a shoot dry weight of inoculated plants significantly higher than that of uninoculated CH3 plants. While SVQ288 and SVQ533 mutants retained the ability to nodulate CH3 accession, the NGRΩ*rhcN* and NGRΩ*ttsI* could no longer induce the formation of nodules (Table 4). By contrast the less selective *G. soja* accession CH4 from Central China nodulated with all the HH103 and NGR234 mutants tested: plants inoculated with SVQ288 and SVQ533 strains formed fewer nodules but shoot dry weight was not significantly altered when compared to CH4 plants inoculated with HH103-1 at 60 days post-inoculation (dpi). *G. soja* CH4 responses to inoculation with NGR*ΩrhcN* or NGR234 were similar, while nodulation and nitrogen-fixation of NGRΩ*ttsI* was significantly reduced on CH4 (Table 4).

As previously described, *S. fredii* strains HH103 Rif^R^ and HH103-1 failed to nodulate *G. soja* CH2. As SVQ288 and SVQ533 mutants deficient in T3SS-dependent secretion gained nodulation, it seems likely that secretion of HH103 Nop complement triggers defensive responses in CH2 plants. Therefore, we tested whether presence of HH103 Rif^R^ would block nodulation of CH2 accession by the proficient symbiont USDA110^T^. When co-inoculated at ratios of 1:1 and 1:10, USDA110^T^ and HH103 Rif^R^ inocula formed at 25 dpi as many as 7.0 ± 1.8 and 7.1 ± 1.4 nodules/plant, respectively. Thus, the presence of HH103 Rif^R^ does not appear to block nodulation of CH2 accession by USDA110^T^.

Competition experiments for nodulation of *G. soja* accessions CH3 and CH4 were carried out between HH103 Rif^R^ and its T3SS-mutant derivatives SVQ533 and SVQ288. On both accessions, SVQ533 and SVQ288 were clearly outcompeted by HH103 Rif^R^ with mutants only occupying 10–15% of the nodules (Table 6).

**Table 6:** Nodule occupancy of *Glycine soja* accessions CH3 and CH4 inoculated with *S. fredii* HH103 and its *ttsI* and *rhcJ* mutants.

*G. soja* accession	Pairs of inoculants	TNN	% of nodule occupancy by mutant SVQ533 or SVQ288
CH3	*S. fredii* HH103 Rif^R^/SVQ533	127	10.2
	*S. fredii* HH103 Str^R^/SVQ288	161	15.5
CH4	*S. fredii* HH103 Rif^R^/SVQ533	156	10.9
	*S. fredii* HH103 Str^R^/SVQ288	207	10.4


*TNN, total number of nodules tested in two independent experiments.*

*In each experiment, 5 jars were inoculated per pair of competitors. Each jar contained 2 plants.*

*YMA supplemented with spectinomycin or neomycin (50 μg/ml) were used to detect the presence of SVQ533 or SVQ288, respectively.*

### Host-Range of *S. fredii-*Symbiotic Plasmid Hybrids on *G. max*, *G. soja* and Other Legumes Normally Nodulated by NGR234

**FIGURE 4:**
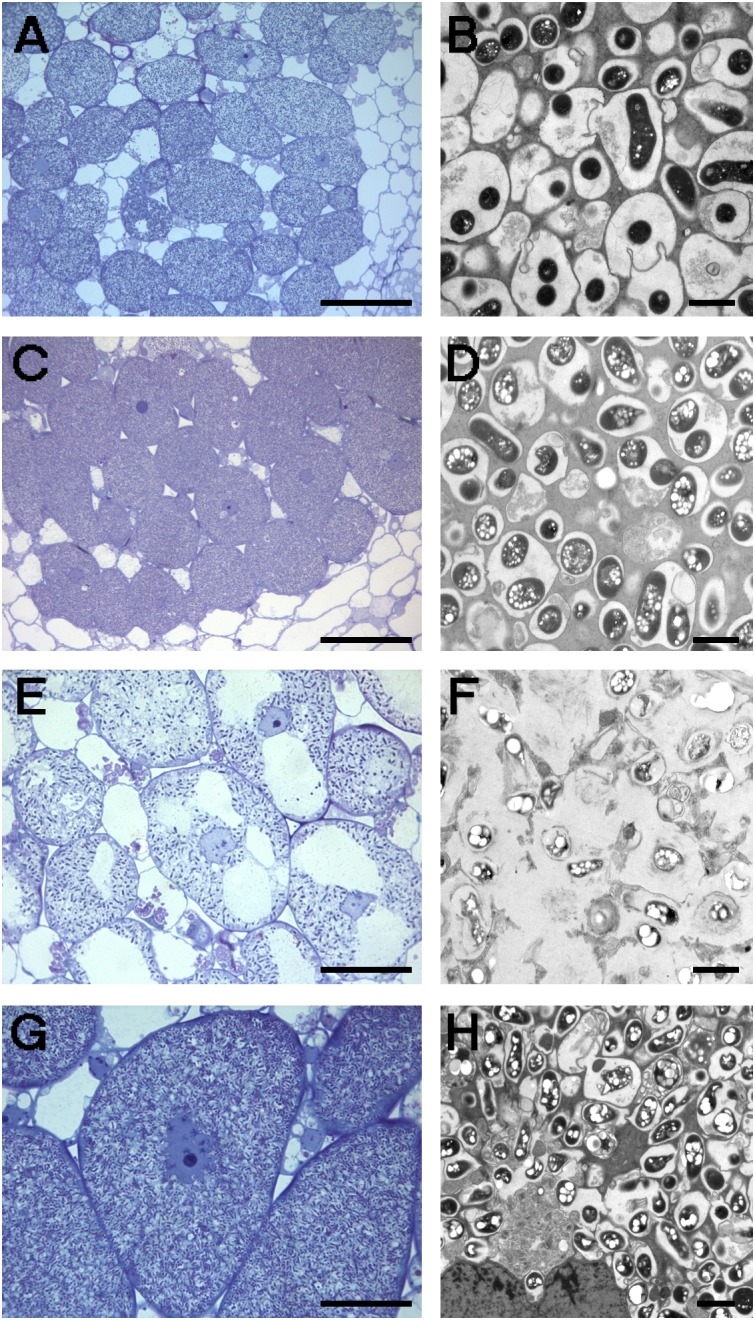
Optical **(A,C,E,G)** and electronic **(B,D,F,H)** microscopy of nodules induced by different *S. fredii* strains in *G. soja* CH4. **(A,B)** HH103 Rif^R^; **(C,D)** NGR234; **(E,F)** NGR234C pSymHH103M; **(G,H)** USDA193C pSymNGR234M. Bars correspond to 100 μm in **(A,C)**, 30 μm in **(E,G)**, and 2 μm in **(B,D,F,H)**.

**FIGURE 5:**
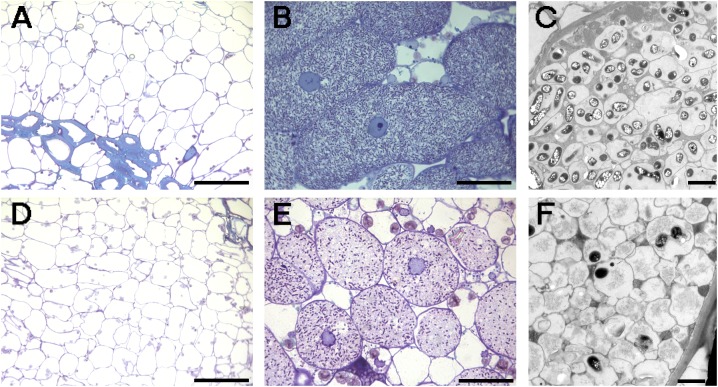
Optical **(A,B,D,E)** and electronic **(C,F)** microscopy of nodules induced by different *S. fredii* strains in soybean cv. Williams 82. **(A)** NGR234; **(B,C)** HH103 Rif^R^; **(D)** USDA193C pSymNGR234M; **(E,F)** NGR234C pSymHH103M. Bars correspond to 100 μm in **(A,D)**, 30 μm in **(B,E)**, and 2 μm in **(C,F)**.

USDA193C and NGR234C transconjugants were tested in nodulation assays with *G. max* cv. Williams 82 and the *G. soja* accession CH4 (Table 7 and Figures 4, 5). Whereas strains NGR234, NGR234-M, and NGR234C (=ANU265) failed to nodulate *G. max* cv. Williams (Table 7), the NGR234C transconjugants carrying plasmid p*Sf*HH103d::Tn*5*-Mob (hereafter pSymHH103M) induced the formation of white ineffective nodules that did not contribute to plant growth. Similarly, USDA193C carrying the symbiotic plasmid p*Sf*NGR234a::Tn*5*-Mob (hereafter pSymNGR234M) only induced the formation of ineffective root outgrowths on *G. max* cv. Williams 82 (Figure 5D). By contrast, *G. soja* CH4 inoculated with USDA193C carrying the symbiotic plasmid pSymNGR234M induced the formation of nitrogen-fixing nodules (Figures 4G,H) and plants did not show symptoms of nitrogen starvation. On the same CH4 host, plants inoculated with the NGR234C pSymHH103M hybrid formed inefficient nodules, however (Table 7).

**Table 7:** *Glycine max* cv. Williams 82 and *G. soja* CH4 responses to inoculation with *S. fredii* pSym-cured derivatives carrying different *S. fredii* pSym plasmids.

Legume	*S. fredii*	Symbiotic plasmid	Number of nodules	Shoot dry-
	inoculant	present	per plant^A,B,C^	weight (mg)
*G. max* cv. Williams	HH103-M	p*Sf*HH103d::Tn*5*-Mob	79.5 ± 6.9^A^ a	2612 ± 424 a
	NGR234	p*Sf*NGR234a	Nod^-B^	645 ± 271 b
	NGR234-M	p*Sf*NGR234a::Tn*5*-Mob	Nod^-B^	700 ± 205 b
	NGR234C	None	Nod^-C^	545 ± 220 b
	NGR234C pSymHH103M	p*Sf*HH103d::Tn*5*-Mob	49.0 ± 16.3^D^ b	480 ± 244 b
	USDA193C	None	Nod^-C^	647 ± 122 b
	USDA193C pSymNGR234M	p*Sf*NGR234a::Tn*5*-Mob	Nod^-B^	508 ± 77 b
	Uninoculated	–	–	507 ± 190 b
*G. soja* CH4	HH103-M	p*Sf*HH103d::Tn*5*-Mob	5.8 ± 2.4^A^ b	239 ± 110 a
	NGR234	p*Sf*NGR234a	3.8 ± 1.1 bc	216 ± 83 ab
	NGR234-M	p*Sf*NGR234a::Tn*5*-Mob	3.2 ± 1.2^A^ c	161 ± 47 c
	NGR234C	None	Nod^-C^	55 ± 18 d
	NGR234C pSymHH103M	p*Sf*HH103d::Tn*5*-Mob	19.9 ± 5.6^D^ a	66 ± 20 d
	USDA193C	None	Nod^-C^	57 ± 13 d
	USDA193C pSymNGR234M	p*Sf*NGR234a::Tn*5*-Mob	3.7 ± 1.0^A^ bc	181 ± 86 bc
	Uninoculated	–	–	64 ± 13 d


*Three Leonard jars containing two (G. max) or four (G. soja) seedlings were inoculated with each bacterial strain. Numbers are mean values ± standard errors. Statistical comparisons among inoculants are shown using ANOVA. For each parameter, data followed by the same small letter are not significantly different at α = 5%.*

*Plants were analyzed 42 days after inoculation.*

*^*A*^Green plants with pink nodules of normal external morphology were formed.*

*^*B*^Yellow plants with macroscopic root outgrowths (MRO) were present.*

*^*C*^Yellow plants and absence of MRO.*

*^*D*^Yellow plants and small white ineffective nodules.*

*Uninoculated controls remained non-nodulated.*

Nodules formed by *S. fredii* inoculants were sectioned, stained with methylene blue, and observed by light microscopy. As expected, *G. soja* CH4 nodules induced by NGR234 contained a high density of symbiosomes (Figures 4C,D), similarly to those induced by HH103 Rif^R^ (Figures 4A,D). By contrast, macroscopic root outgrowths (MRO) formed in *G. max* cv. Williams 82 inoculated with NGR234 were devoid of bacteria inside the cells and in the intercellular spaces. Clusters of plant cells showing thick walls were observed (Figure 5A). Ineffective nodules formed by *G. soja* CH4 and *G. max* cv. Williams 82 inoculated with NGR234C carrying pSymHH103M contained many but poorly infected cells as in senescent nodules (Figures 4E, 5E). Electron micrographs confirmed that *G. soja* CH4 and *G. max* cv. Williams 82 nodules occupied by NGR234C carrying pSymHH103M were senescent, with nodule cells containing degraded bacteroids at 42 dpi (Figures 4F, 5F). At this same time point, *G. soja* nodules infected with NGR234 (Figures 4C,D) and *G. max* nodules occupied by HH103 Rif^R^ (Figures 5B,C) did not show visible symptoms of senescence.

**FIGURE 6:**
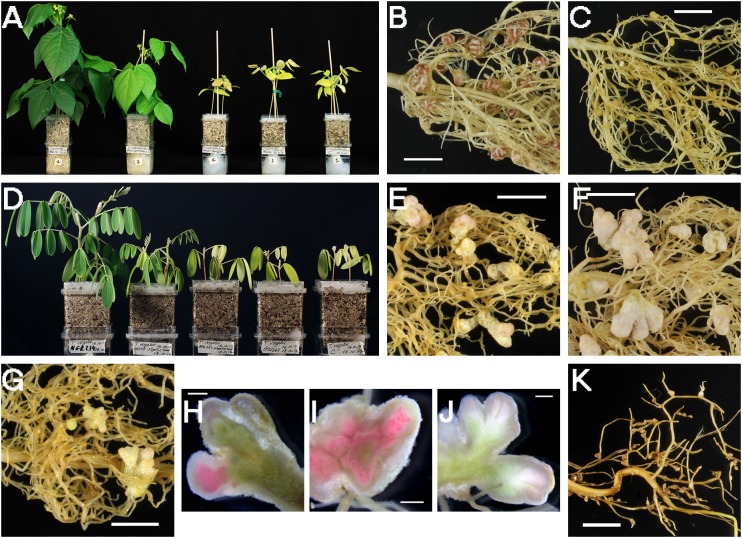
Plant responses of three host legumes of *S. fredii* NGR234, *Vigna radiata* **(A–C)**, *Tephrosia vogelii* **(D–J)** and *Leucaena leucocephala* **(K)**, with different *S. fredii* strains. **(A)** Phenotypes of strains on mung bean (*V. radiata*) at 42 dpi. From left to right, plants inoculated with NGR234, HH103-M, NGR234C pSymHH103M, NGR234C, and uninoculated control. **(B)** Nodules induced by HH103-M in *V. radiata* roots (42 dpi). **(C)** Pseudonodules induced by NGR234C pSymHH103M in *V. radiata* roots (42 dpi). **(D)** Phenotypes of strains on *T. vogelii* at 58 dpi. From left to right, plants inoculated with NGR234, HH103-M, NGR234C pSymHH103M, NGR234C, and uninoculated controls. **(E–J)** Nodules induced by HH103-M **(E,H)**, NGR234 **(F,I)**, and NGR234C pSymHH103M **(G,J)** in *T. vogelii* roots (58 dpi). **(K)** Abnormal root outgrowths on *L. leucocephala* 81 dpi with NGR234C pSymHH103M. Bars correspond to 1 cm in **(B,C,E–G,K)**, and 1 mm in **(H–J)**.

Further nodulation assays were carried out to investigate the capacity of the symbiotic plasmid donor *S. fredii* HH103-M and the resulting NGR234C pSymHH103M hybrid strain to nodulate with three different legumes that form indeterminate (*L. leucocephala* and *T. vogelii*) or determinate (*V. radiata*) nodules with NGR234. *V. radiata* cv. King and *T. vogelii* plants inoculated with HH103-M formed nodules (Figures 6B,E), although both symbioses were less effective than those established with NGR234 (Table 8 and Figures 6A,D). *L. leucocephala* roots inoculated with HH103-M or NGR234C carrying pSymHH103M formed macroscopic root outgrowths (MRO) that did not contribute to plant growth (Table 8 and Figure 6K). Although *V. radiata* plants inoculated with NGR234 or HH103-M formed nitrogen-fixing nodules, the hybrid strain NGR234C carrying pSymHH103M only formed ineffective nodules (Figure 6C) and the color and development of plants were similar to those of the uninoculated control (Figure 6A). Despite the number of nodules formed by *T. vogelii* roots inoculated with NGR234 or HH103-M being similar (Table 8 and Figures 6E,F), the former was much more effective in supporting plant growth (Figure 6D). In contrast to the fully proficient indeterminate nodules induced by NGR234 on *T. vogelii*, (Figure 6I), those formed upon inoculation with HH103-M appeared mostly senescent with presence of leghemoglobin restricted to small sections of these nodules (Figure 6H). Hybrid strain NGR234C pSymHH103M induced the formation of ineffective nodules on *T. vogelii* roots (Table 8 and Figures 6G,J).

**Table 8:** *Leucaena leucocephala*, *T. vogelii*, and *V. radiata* responses to inoculation with a NGR234 pSym-cured derivative (ANU265) carrying the symbiotic plasmid of HH103 Rif^R^ tagged with Tn*5*-Mob.

Legume	Inoculant	Number of	Shoot dry
		nodules	weight (mg)
*L. leucocephala*	Uninoculated	0.0	102 ± 8
	NGR234C	0.0	101 ± 40
	NGR234	36 ± 10	975 ± 259
	HH103-M	0.0	97 ± 27
	NGR234C pSymHH103M	0.0	120 ± 35
*T. vogelii*	Uninoculated	0.0	152 ± 24
	NGR234C	0.0	107 ± 26
	NGR234	18 ± 3	1171 ± 220
	HH103-M	13 ± 7	312 ± 106
	NGR234C pSymHH103M	5 ± 2	118 ± 37
*V. radiata*	Uninoculated	0.0	210 ± 47
	NGR234C	0.0	182 ± 40
	NGR234	162 ± 21	2919 ± 367
	HH103-M	57 ± 12	1564 ± 241
	NGR234C pSymHH103M	Only pseudonodules	175 ± 33


*Numbers refer to mean values ± standard errors per plant of three Magenta jars containing two plants. Determinations were carried out 42 (V. radiata), 58 (T. vogelii), and 81 (L. leucocephala) days after inoculation.*

## Discussion

In the last two decades, different reports describing the geographical occurrence and distribution of slow- and fast-growing soybean rhizobia have been reported. A general conclusion from these studies is that soil pH appears to determine the prevalence of soybean bradyrhizobia (slow growers) or soybean sinorhizobia (fast growers). The most detailed report (Tian et al., 2012) confirmed that soybean bradyrhizobia populations are more abundant in acidic soils (Northern and Southern China), while soybean sinorhizobia are predominant in alkaline soils of Central China (Hebei, Shanxi, Henan, and Shandong provinces). The central province of Shaanxi was not included in this study. Recent work from India also suggests that *Bradyrhizobium* strains are very abundant as nodulators of a number of native legumes in acidic soils, whereas *Sinorhizobium* are more prevalent in neutral-alkaline soils (Ojha et al., 2017; Sankhla et al., 2017; Rathi et al., 2018).

In this work we have investigated the symbiotic capacity of wild soybeans from Northern and Central China to associate with *B. diazoefficiens* USDA110^T^, *B. elkanii* USDA76^T^, and *S. fredii* HH103 Rif^R^, three well-known soybean-rhizobia strains whose genome have been fully sequenced and deposited in public databases. These soybean-rhizobia were also tested with wild soybeans from Russia, South Korea, and Japan, which are the only geographical areas outside China in which *G. soja* was so far reported.

Symbiotic nitrogen fixation of wild soybean accessions from Russia, South Korea, Japan, and Northern China inoculated with HH103 Rif^R^ was not effective enough to support appropriate plant growth in the absence of nitrogen fertilizers (Table 2 and Supplementary Tables S5, S8). These results indicate that wild soybeans from geographical areas in which fast-growing soybean-rhizobia populations are absent, or at very low levels, appear to have a low symbiotic affinity for HH103 Rif^R^. Moreover, two accessions from the Jilin province, CH2 and CH12, even failed to nodulate with this strain, indicating that in these two accessions, symbiotic incompatibility was triggered by yet unknown molecular determinants. All the *G. soja* accessions from Russia, South Korea, Japan, and Northern China formed nitrogen-fixing nodules with USDA110^T^ and USDA76^T^, the former generally being more effective than the latter (Table 2 and Supplementary Tables S3, S4, S6, S7). Interestingly, *B. japonicum* and *B. diazoefficiens* appear to be more abundant than *B. elkanii* in the Northern provinces of China (Figure 1; Tian et al., 2012). Nodules formed by HH103 Rif^R^ in six wild soybeans from the Shanxi province (Central China, alkaline soils) fixed enough nitrogen to support plant growth (Supplementary Tables S5, S8). All these results suggest that, at least in Northern and Central China, there is a correlation between symbiotic fitness of wild soybeans from a particular geographical area and the relative abundance of the different soybean rhizobia genera and species dwelling in soils of that specific zone. Concomitantly, *G. soja* accessions from Northern China are generally more effective with USDA110^T^ than with USDA76^T^.

In Central China *S. fredii* indigenous populations are more abundant than bradyrhizobia populations (Tian et al., 2012). Some *G. soja* accessions from Shanxi (CH4, CH24, and CH25) were found to be more effective with HH103 Rif^R^ than with USDA110^T^, whereas the CH21 and CH23 accessions favored USDA110^T^. Thus, the possible correlation between the relative abundance of specific soybean-rhizobia and symbiotic fitness with local *G. soja* accessions from Central China is not as clear as in the case of plant-rhizobia combinations from the Northern provinces. To our knowledge, there are no reports describing the soybean indigenous populations of soils from Shaanxi province. Some accessions (e.g., CH17 and CH20) were more effective with HH103 Rif^R^ but others showed higher REI values with USDA110^T^ and USDA76^T^ (such as CH16 and CH18). Together these results indicate that Shanxi and Shaanxi provinces are promising places for finding wild soybeans showing high nitrogen-fixation capacities with *S. fredii* strains. Central China also appears to be promising for finding wild soybeans showing good symbiotic capacities with slow- and fast-growing soybean rhizobia, which is in contrast with *G. soja* accessions from Northern China, that appear to be only effective with bradyrhizobia.

Competition studies showed that USDA110^T^ was more competitive than USDA76^T^ to nodulate accessions CH2 and CH3 (Northern China) but less competitive with accession CH4 (Central China). These results fit well with the fact that soils from Northern China contain *B. japonicum* and *B. diazoefficiens* strains but are apparently devoid of *B. elkanii* populations, whereas soils of Central China are rather populated by *B. elkanii* instead of *B. japonicum* or *B. diazoefficiens* strains (Tian et al., 2012).

*Sinorhizobium fredii* NGR234 shows the broadest nodulation host-range ever described (Pueppke and Broughton, 1999). Strikingly, and unlike most *S. fredii* strains known, NGR234 is unable to nodulate soybean (*G. max*). The fact that some wild soybeans from Central China formed effective symbioses with HH103 Rif^R^ prompted us to investigate whether NGR234 could also form nitrogen-fixing nodules with any of these *G. soja* accessions. In fact, NGR234 formed nitrogen-fixing nodules with at least 6 different wild soybean accessions form Shaanxi or Shanxi provinces. Plant tests carried out in Spain, Switzerland, and China proved that NGR234 consistently nodulated several *G. soja* accessions, regardless of experimental settings and plant growth conditions used. To our knowledge, this is the first report describing the formation of nitrogen-fixing nodules in *G. soja* plants inoculated with *S. fredii* NGR234. Such a robust symbiotic phenotype on *G. soja* make attractive further searches for finding amongst the *G. max* varieties grown by Chinese farmers in Central China those capable of forming nitrogen-fixing nodules with NGR234.

It is well known that formation of symbiotic root nodules in soybean is controlled by several host genes referred to as *Rj* (*rj*) genes and it has been repeatedly reported that *S. fredii* T3SS plays important roles in determining symbiotic compatibility with *G. max* cultivars and other legume species (Hayashi et al., 2012; Staehelin and Krishnan, 2015; López-Baena et al., 2016; Yasuda et al., 2016). Here, we also tested whether the T3SS of HH103 Rif^R^ and NGR234 strains influenced nodulation of wild soybeans. Results confirmed that mutations blocking Nop-secretion significantly impacted symbiosis with a number of *G. soja* accessions (Table 4). For example, CH2 accession from Northern China formed nodules with NGR234 but not with HH103 Rif^R^. Conversely, while mutants of NGR234 with deficient T3SS (strains NGRΩ*ttsI* and NGRΩ*rhcN*) lost their capacity to nodulate CH2 the HH103 mutants in *ttsI* and *rhcJ* gained nodulation on this accession. These results indicate that one or more of the five Nop secreted by NGR234 promote(s) CH2 nodulation, while the combination of Nop secreted by HH103 Rif^R^ prevents CH2 nodulation. Although the sequences of the Nop shared by HH103 Rif^R^ and NGR234 are nearly identical, there are some differences in the repertoire of T3SS effectors secreted by both strains (López-Baena et al., 2016). For example, HH103 Rif^R^ carries *nopI* and *nopD* genes, which are absent from the NGR234 genome. By contrast, NGR234 genome includes a copy of the *nopJ* gene that is absent from HH103 Rif^R^. These three genes are therefore good candidates for determining symbiotic specificity on CH2. On the CH3 (from Northern China) and CH4 (Central China) accessions, HH103 Rif^R^ and the *ttsI* (SVQ533) or *rhcJ* (SVQ288) derivative mutants retained nodulation. Shoot dry-weight of CH3 plants inoculated with SVQ533 was significantly lower than for plants inoculated with the parent strain (Table 4). Mutations in *ttsI* and *rhcN* of NGR234 totally abolished nodulation on both CH3 and CH2 accessions, however. These phenotypes suggest that NopJ (the only T3SS-effector not secreted by HH103 Rif^R^) is a prime candidate for promoting nodulation of CH2 and CH3 by NGR234. Since CH4 plants responded in the same manner to inoculation with NGR234 or NGRΩ*rhcN*, T3SS and its panel of effectors don’t play a significant role during nodulation of this accession. That a significant reduction in all phenotypic parameters monitored occurred when CH4 plants were inoculated with NGRΩ*ttsI*, indicate that a TtsI-regulated function other than T3SS plays an important role during the NGR234-CH4 interaction, however. Together these results confirm that T3SS play important roles (either positively or negatively) during symbioses between *S. fredii* strains and wild soybean accessions. Also, the different effect that the studied T3SS mutations have in the symbiotic behavior with the different *G. soja* accessions tested suggests that these *G. soja* accessions might have different *Rj* (*rj*) genotypes, although further research is necessary for clarifying this issue.

There is a theoretically alternative way to carry out screenings aimed to isolate new *S. fredii* strains showing altered symbiotic capacities with soybean and other legumes: the construction of *S. fredii* hybrid strains (chimeras) by intraspecific transfer of symbiotic plasmids. The simplest way for constructing such chimeras is by transferring *S. fredii* symbiotic plasmids to pSym-cured derivatives of other *S. fredii* strains. If the symbiotic properties of some of these combinations (*S. fredii* genome background/*S. fredii* pSym) appear to be interesting, the analysis of the hybrids will be easier if the genome sequences of the donor and recipient strains are already known. This would be the situation for hybrids constructed between *S. fredii* strains NGR234 and HH103 Rif^R^. Following this approach, we transferred the symbiotic plasmid of HH103 Rif^R^ to strain NGR234C, a pSym-cured derivative of NGR234. The symbiotic plasmid of NGR234 was marked with transposon Tn*5*-Mob and transferred to *S. fredii* USDA193C, a pSym-cured derivative of *S. fredii* USDA193 (unfortunately, a pSym-cured derivative of HH103 Rif^R^ is not available). USDA193 forms nitrogen-fixing nodules with Asiatic soybean cultivars but root-swellings and/or ineffective nodules with American soybeans (Keyser et al., 1982).

NGR234 does not effectively nodulate the American soybean cultivar Williams 82. Although Trinick (1980) reported that NGR234 induce Fix^+^ nodules on *G. max*, the cultivar employed in that study was not specified. Balatti et al. (1995) showed that NGR234 only induces the formation of small, irregular swellings on the roots of 89 different varieties of soybean, including McCall (restriction genotype *Rfg1*) and Dunfield (restriction genotype *Rj4*). Pueppke and Broughton (1999) also found that NGR234 was ineffective with one Asiatic (Peking) and two American (McCall and Preston) varieties of soybean. So, it can be concluded that NGR234 is ineffective with the vast majority of soybean cultivars tested so far.

The *Rj* genotype of soybean Williams 82 is *Rfg1*, which restricts nodulation by *S. fredii* USDA257 (reviewed by Hayashi et al., 2012). This *Rj* genotype is shared by other American varieties such as McCall. This restriction is most probably due to the type three associated effectors secreted by USDA257, since mutants of this strain in this secretion system gain the ability to nodulate American soybeans (reviewed by Staehelin and Krishnan, 2015; López-Baena et al., 2016). Most probably, this is also the reason why other *S. fredii* strains such as USDA192 or USDA193 also fail in nodulating American soybeans. However, the story appears to be more complex since strain HH103, which also possesses a functional symbiotic type three secretion system (T3SS), naturally nodulates American soybeans (including Williams 82) and mutants negatively affected in its T3SS undergo a partial impairment in their interactions with these soybeans (de Lyra et al., 2006).

The chimeric strain NGR234C pSymHH103M formed nodules that did not fix nitrogen with soybean Williams 82, so that the shoot dry-weight of plants inoculated with the hybrid strain was equal to that of the uninoculated control (Table 7). Thus, the presence of the pSymHH103M, which is the symbiotic plasmid of a strain (HH103-M) that effectively nodulates Williams 82, is not enough to enable NGR234C to form nitrogen-fixing nodules with this *G. max* cultivar. Three possible explanations can be formulated: (i) some genes essential for effective soybean nodulation are neither present in NGR234C nor provided by pSymHH103M; (ii) some of the NGR234C genes block nodule development; and (iii) the combination of symbiotic signals produced by the resulting chimeric strain is not suitable for effective nodulation of *G. max* cultivar Williams 82. Although the NGR234C pSymHH103M transconjugant nodulated *G. soja* CH4, nodules that were formed did not contribute to plant growth (Table 7). Microscopy studies showed that peribacteroid membranes were disrupted or missing, as is often the case when nodules senesce (Figure 4F). This result, that was unexpected as both NGR234 and HH103 Rif^R^ strains were capable to form proficient nodules on this accession, suggest the combination of symbiotic signals produced by the hybrid strain might not be suitable for making nitrogen-fixing nodules on CH4 accession. For instance, the hybrid must produce the HH103 Rif^R^ complement of nodulation factors and secreted Nop since genes involved in these symbiotic functions are carried by the symbiotic plasmid. The hybrid, however, should produce the KPS synthesized by NGR234 because *rkp* genes are carried by pNGR234b (Schmeisser et al., 2009). Possibly, LPS of the NGR234C pSymHH103M hybrid should differ from those of NGR234 and HH103 Rif^R^ as most genes involved in LPS biosynthesis are chromosome born. However, some genes required for the biosynthesis of the flavonoid-inducible rhamnan modification of the NGR234 LPS (Marie et al., 2004) are encoded by p*Sf*NGR234a and thus neither present in NGR234 nor provided by plasmid pSymHH103M (Vinardell et al., 2015).

The *S. fredii* strains NGR234 and HH103-M nodulated *T. vogelii*, the former being more effective (Table 8). *T. vogelii* plants inoculated with the hybrid NGR234C pSymHH103M showed reduced nodulation in comparison to the parental strains and the nodules formed did not support plant growth (Table 8). These results suggest again that the combination of symbiotic signals produced by the hybrid is less appropriate than that produced by each parental strains. The failure of hybrid NGR234C pSymHH103M to nodulate *L. leucocephala* (Table 8) could be due to the absence of a functional *nodS* gene, which is required for nodulation of this legume (Lewin et al., 1990). The *nodS* gene is located in the symbiotic plasmid of NGR234 (missing in NGR234C) and the *nodS* present in pSymHH103 is not functional (Vinardell et al., 2015).

The hybrid USDA193C carrying the symbiotic plasmid of NGR234 (USDA193C pSymNGR234M) failed to nodulate *G. max* cv. Williams 82 but formed nitrogen-fixing nodules with *G. soja* CH4. Thus, the symbiotic properties of the two hybrids studied (NGR234C pSymHH103M and USDA193C pSymNGR234M) are different. We could expect that new combinations would produce “new” *S. fredii* strains in which the symbiotic interaction with a particular legume could be positive or negatively affected or without any noticeable change. The genetic information already available about *S. fredii* HH103 Rif^R^ and NGR234 strains, together with the structural information of their Nodulation factors, surface polysaccharides and nodulation outer proteins, might allow the prediction of the symbiotic signals produced by the hybrid strains. Taking the advantage that *S. fredii* strains nodulate many different legumes, the study of *S. fredii* hybrids could provide valuable information about the relative importance of particular symbiotic signals, or combination of signals, for the bacterial interaction with a wide range of legumes.

## Author Contributions

FT-V, XP, JR-S, and JV conceived and designed the experiments. All authors performed the experiments. FT-V, SA-J, XP, JR-S, FL-B, and JV analyzed the data. FT-V, XP, JR-S, and JV contributed with reagents, materials, and analysis tools. FT-V, XP, FL-B, JR-S, and JV wrote the paper. All authors read and approved the final manuscript.

## Conflict of Interest Statement

The authors declare that the research was conducted in the absence of any commercial or financial relationships that could be construed as a potential conflict of interest.
