# Supplementary material for: Sinorhizobium fredii Strains HH103 and NGR234 Form Nitrogen Fixing Nodules With Diverse Wild Soybeans (Glycine soja) From Central China but Are Ineffective on Northern China Accessions
Source: Front Microbiol. 2018 Nov 21;9:2843. doi: 10.3389/fmicb.2018.02843 (PMC6258812; doi:10.3389/fmicb.2018.02843)
Supplement: Supplementary file 1 [file Table_1.docx]

**Supplementary Table S1.** Bacterial strains and plasmids.

| **Strain or plasmid** | **Derivation and relevant properties** | **Source or Reference** |
| --- | --- | --- |
| ***Sinorhizobium fredii*** |  |  |
| NGR234 | *S. fredii* strain isolated from nodules of *Lablab* *purpureus*, Rif^R^ | Trinick, 1980 |
| NGRΩ*rhcN* | NGR234 carrying an Omega-interposon insertion in *rhcN* | Viprey *et al*., 1998 |
| NGRΩ*ttsI* | NGR234 carrying an Omega-interposon insertion in *ttsI* | Viprey *et al*., 1998 |
| NGR234-D17  (=NGR234-M) | NGR234 carrying a Tn*5*-Mob insertion in the symbiotic plasmid p*Sf*NGR234a | This work |
| ANU265  (=NGR234C) | pSym-cured derivative of NGR234(Rif^R^) | Morrison *et al*., 1983 |
| ANU265 p*Sf*HH103d::Tn*5*-Mob  (=NGR234C pSymHH103M) | ANU265 transconjugant carrying the symbiotic plasmid p*Sf*HH103d::Tn*5*-Mob | This work |
| HH103 | Wild type strain | Dowdle and Bohlool. 1985 |
| HH103-1 | Spontaneous streptomycin resistant derivative of HH103 | Buendía-Clavería *et al*., 1989a |
| SVQ288 | HH103-1 *rhcJ*::Tn*5*::*lacZ* | Vinardell *et al*., 2004a |
| HH103 Rif^R^  (=SVQ269) | Spontaneous rifampicin resistant derivative of HH103 | Madinabeitia *et al*., 2002 |
| SVQ533 | HH103-Rif^R^ carrying an Omega-interposon insertion in *ttsI*. | Lopez-Baena *et al*., 2008 |
| HH103-M | HH103-Rif^R^ carrying a Tn*5*-Mob insertion in the symbiotic plasmid p*Sf*HH103d::Tn*5*-Mob, Nm^R^ | Vinardell *et al*., 2004b |
| USDA193 | Wild-type strain | Keyser *et al*., 1982 |
| USDA193-Str^R^-Spc^R^  (=AB268) | Spontaneous streptomycin and spectinomycin resistant derivative of USDA193 | Buendía-Clavería *et al*., 1989b |
| USDA193C  (=AB359) | USDA193 Str^r^ Spc^r^ cured of the symbiotic plasmid p*Sf*USDA193a::Tn*5*-Mob | Buendía-Clavería *et al*., 1989b |
| USDA193C p*Sf*NGR234a::Tn*5*-Mob  (=USDA193C pSymNGR234M) | USDA193C carrying the symbiotic plasmid p*Sf*NGR234a::Tn*5*-Mob | This work |
| USDA193C p*Sf*HH103d::Tn*5*-Mob  (=USDA193C pSymHH103M) | USDA193C carrying the symbiotic plasmid p*Sf*HH103d::Tn*5*-Mob | Vinardell *et al*., 2004b |
|  |  |  |
| ***Agrobacterium tumefaciens*** |  |  |
| GMI9023 Cm^R^  (=AB274) | Cm^R^ Rif^R^ Str^R^ pTi-cured derivative of *A. tumefaciens* C58 | Cubo *et al*., 1988 |
|  |  |  |
| ***Bradyrhizobium diazoefficiens*** |  |  |
| USDA110^T^ | Wild type strain, highly effective with different American soybean cultivars | Sadowsky *et al*., 1987; Delamuta *et al*., 2013 |
| ***Bradyrhizobium elkanii*** |  |  |
| USDA76^T^ | Wild type strain, highly effective with different American soybean cultivars | Kuykendall *et al*., 1992 |
| ***Escherichia coli*** |  |  |
| S17-1 | 294 Rec^-^ chromosomally integrated RP4 derivative, Tp^R^ Str^R^ | Simon *et al*., 1983 |
| HB101 | Restriction-minus, *recA* background, Str^R^ | Boyer and Roulland-Dossoix, 1969 |
|  |  |  |
| ***Plasmids*** |  |  |
| pRK2013 | ColE1 replicon containing the *tra* genes of RK2, Nm^R^ Km^R^ | Figurski and Helinski, 1979 |
| pSUP5011 | pBR325::Tn*5*-Mob | Simon *et al*., 1983 |
| p*Sf*HH103d::Tn*5*-Mob  (= pSymHH103M) | *S. fredii* HH103 symbiotic plasmid carrying a Tn*5*-Mob insertion | Vinardell *et al*., 2004b |
| p*Sf*NGR234a::Tn*5*-Mob  (= pSymNGR234M) | *S. fredii* NGR234 symbiotic plasmid carrying a Tn*5*-Mob insertion | This work |
| pMUS248 | IncQ KmR^r^ plasmid in which the *tet* gene is under the control of the pRL1JI *nodA* promoter | Vinardell *et al*., 1993 |
| pMUS262 | Km^S^ Gm^R^ derivative of pMUS248 | Vinardell *et al*., 1993 |

**References:**

Boyer, H. B., and Roulland-Dussoix, D. (1969). A complementation analysis of the restriction and modification of DNA in *Escherichia coli*. *J. Mol. Biol.* 4, 459-472.

Buendía-Clavería, A. M., Chamber, M. M., and Ruiz-Sainz, J. E. (1989a). A comparative study of the physiological characteristics, plasmid content and symbiotic properties of different *Rhizobium fredii* strains. *Syst. Appl. Microbiol.* 12, 203-209.

Buendía-Clavería, A. M., Romero, F., Cubo, T., Perez-Silva, J., and Ruiz-Sainz, J. E. (1989b). Inter and intraspecific transfer of a *Rhizobium* *fredii* symbiotic plasmid: Expression and incompatibility of symbiotic plasmids. *Syst. Appl. Microbiol.* 12, 210-215.

Cubo, M. T., Buendía-Clavería, A. M., Beringer, J. E., and Ruiz-Sainz, J. E. (1988). Melanin production by *Rhizobium* strains. *Appl. Environ. Microbiol.* 54, 1812-1817.

Delamuta, J. R., Ribeiro, R. A., Ormeño-Orrillo, E., Melo, I. S., Martínez-Romero, E., and Hungria, M. (2013). Polyphasic evidence supporting the reclassification of *Bradyrhizobium japonicum* group Ia strains as *Bradyrhizobium diazoefficiens* sp. nov. *Int. J. Syst. Evol. Microbiol.* 63, 3342-3351.

Dowdle, S. F., and Bohlool, B. B. (1987). Intra- and inter-specific competition in *Rhizobium fredii* and *Bradyrhizobium japonicum* as indigenous and introduced organisms. *Can. J. Microbiol.* 33, 990-995.

Figurski, D. H., and Helinski, D. R. (1979). Replication of an origin containing derivative of plasmid RK2 dependent on a plasmid function provided *in trans*. *Proc. Natl. Acad. Sci. USA* 76, 1648–1652.

Keyser, H. H., Bohlool, B. B., Hu, T. S., and Weber, D. F. (1982). Fast-growing rhizobia isolated from root nodules of soybean. *Science* 215, 1631-1632.

Kuykendall, L.D., Saxena, B., Devine, T.E., and Udell, S.E. (1992). Genetic diversity in *Bradyrhizobium japonicum* Jordan 1982 and a proposal for *Bradyrhizobium elkanii* sp. nov. *Can. J. Microbiol.* 38, 501-505.

López-Baena, F. J., Vinardell, J. M., Pérez-Montaño, F., Crespo-Rivas, J. C., Bellogín, R. A., Espuny, M. R., and Ollero, F. J. (2008). Regulation and symbiotic significance of nodulation outer proteins secretion in *Sinorhizobium fredii* HH103. *Microbiology-SGM* 154, 1825-1836.

Madinabeitia, N., Bellogín, R. A., Buendía-Clavería, A. M., Camacho, M., Cubo, T., Espuny, M. R., *et al*. (2002). *Sinorhizobium fredii* HH103 has a truncated *nolO* gene due to a -1 frameshift mutation that is conserved among other geographically distant *S. fredii* strains. *Mol. Plant-Microbe Interact.* 15, 150-159.

Morrison, N. A., Hau, C. Y., Trinick, M. J., Shine, J., and Rolfe, B. G. (1983). Heat curing of a Sym plasmid in a fast-growing *Rhizobium* sp. that is able to nodulate legumes and the nonlegume *Parasponia* sp. *J. Bacteriol.* 153, 527-531

Sadowsky, M. J., Bohlool, B. B., and Keyser, H. H. (1987). Serological relatedness of *R. fredii* to other rhizobia and to the bradyrhizobia. *Appl. Environ. Microbiol.* 53, 1785-1789.

Simon, R., Priefer, U., and Pühler, A. (1983). A broad host range mobilization system for *in vivo* genetic engineering: transposon mutagenesis in Gram-negative bacteria. *J. Biotechnol.* 1, 784-791.

Trinick, M. J. (1980). Relationships amongst the fast-growing *Rhizobium* of *Lablab* *purpureus*, *Leucarena leucocephala*, *Mimosa* sp., *Acacia farnesiana*, and *Sesbania grandiflora* and their affinities with other *Rhizobium* groups. *J. Appl. Bacteriol.* 49:39-53.

Vinardell, J. M., Buendía-Clavería, A. M., and Ruiz-Sainz, J. E. (1993). A method for the positive selection of spontaneous *Rhizobium* mutants showing transcriptional activation of nodulation genes in the absence of *nod*-inducers. *Mol. Plant Microbe Interact.* 6, 782-785.

Vinardell, J.M., López-Baena, F. J., Hidalgo, A., Ollero, F. J., Bellogín, R., Espuny, M. R. *et al*. (2004b). The effect of FITA mutations on the symbiotic properties of *Sinorhizobium fredii* varies in a chromosomal-background-dependent manner. *Arch. Microbiol.* 181, 144-154.

Vinardell, J. M., Ollero, F. J., Hidalgo, A., López-Baena, F. J., Medina, C., Ivanov-Vangelov, K., *et al*. (2004a). NolR regulates diverse symbiotic signals of *Sinorhizobium* *fredii* HH103. *Mol. Plant Microbe Interact.* 17, 676–685.

Viprey, V., del Greco, A., Golinowski, W., Broughton, W. J., and Perret, X. (1998). Symbiotic implications of type III protein secretion machinery in Rhizobium. *Mol. Microbiol.* 28, 1381-1389.
