# Supplementary material for: Sinorhizobium fredii Strains HH103 and NGR234 Form Nitrogen Fixing Nodules With Diverse Wild Soybeans (Glycine soja) From Central China but Are Ineffective on Northern China Accessions
Source: Front Microbiol. 2018 Nov 21;9:2843. doi: 10.3389/fmicb.2018.02843 (PMC6258812; doi:10.3389/fmicb.2018.02843)
Supplement: Supplementary file 2 [file Table_2.DOCX]

**Supplementary Table S2.** Geographical origin and maturity group of *G. soja* accessions from Russia, South Korea, and Japan.

| ***G. soja* accession number in IFAPA collection** | **Other accession numbers^A^** | **Region or province** | **Locality** | **Maturity group** | **GPS coordinates** | |
| --- | --- | --- | --- | --- | --- | --- |
|  |  |  |  |  | **Latitude** | **Longitude** |
| **Wild soybeans from Russia** | | | | | | |
| R1 | 81762 | Amur | --- | II | --- | --- |
| R2 | 522195 | Primorye | Far East | II | 44º 60’ | 134º 60’ |
| R3 | 522116 | Primorye | Far East | III | 44º 60’ | 134º 60’ |
| R4 | 522217 | Primorye | Far East | II | 44º 60’ | 134º 60’ |
| R5 | 578340 | Khabarovsk | Habarovsk | I | 48º 29’ | 135º 7’ |
| **Wild soybeans from South Korea** | | | | | | |
| K1 | 407196 | Kangwon | Hongcheon Gun | IV | 37º 40’ | 127º 52’ |
| K2 | 407217 | Chungchong Puk | Jungweon Gun | IV | 36º 57’ | 127º 51’ |
| K3 | 407275 | Kyonggi | Siheung Gun | IV | 37º 25’ | 126º 59’ |
| K4 | 424004A | Kyonggi | Sung-gok Ri | II | 37º 25’ | 126º 47’ |
| K5 | 562565 | Cholla Puk | Osu Ri | IV | 35º 32’ | 127º 20’ |
| **Wild soybeans from Japan** | | | | | | |
| J1 | 366122 | Fukushima | Aizutakada | IV | 37º 27’ | 139º 50’ |
| J2 | 366123 | Iwate | Morioka | IV | 39º 42’ | 41º 15’ |
| J3 | 507581 | Aomori | Kuroishi | III | 40º 38’ | 140º 36’ |
| J4 | 507592 | Akita | Nishisenboku | IV | 39º 42’ | 140º 43’ |
| J5 | 514674 | Hokkaido | Amanogawa River | III | 42º 45’ | 142º 44’ |

^A^ The accession numbers listed in the column are the plant introduction numbers given by the United States Department of Agriculture (USDA) collection.
