# Supplementary material for: Sinorhizobium fredii Strains HH103 and NGR234 Form Nitrogen Fixing Nodules With Diverse Wild Soybeans (Glycine soja) From Central China but Are Ineffective on Northern China Accessions
Source: Front Microbiol. 2018 Nov 21;9:2843. doi: 10.3389/fmicb.2018.02843 (PMC6258812; doi:10.3389/fmicb.2018.02843)
Supplement: Supplementary file 3 [file Table_3.docx]

**Table S3.** Symbiotic responses of *Glycine soja* accessions from Korea, Japan, Russia, and China to inoculation with *B. diazoefficiens* USDA110^T^.

| **Accession** | **Number of nodules** | **Dry weight of nodules (mg)** | **Shoot dry-weight (mg) of plants** | | | **REI**  **(%)** |
| --- | --- | --- | --- | --- | --- | --- |
|  |  |  | **Inoculated** | **Uninoculated** | **N-fertilized** |  |
| **Wild-soybean accessions from Korea** | | | | | | |
| K1 | 45.0±14.2 a | 89.7± b | 1325±15 f | 112±7 g | 4780±125 cd | 26.0 |
| K2 | 114.3±15.8 a | 366.0±18.6 a | 5203±391 c | 121±21 g | 5293±292 c | 98.3 |
| K3 | 110.3±43.8 a | 326.7±84.3 a | 3427±1015 e | 184±22 g | 6430±300 b | 51.9 |
| K4 | 83.7±30.0 a | 292.7±55.8 a | 3943±423 de | 106±12 g | 6520±110 b | 59.8 |
| K5 | 96.5±24.5 a | 338.5±69.5 a | 6690±290 ab | 306±45 fg | 7680±662 a | 86.6 |
| LSD(p<0.05) | n.s. | 169.5 | 1063 | | |  |
| **Wild-soybean accessions from Japan** | | | | | | |
| J1 | 58.3±5.4 ab | 145.3±13.9 c | 2090±259 ef | 187±52 g | 5630±358 a | 35.0 |
| J2 | 95.0±10.5 a | 277.7±25.3 a | 3030±127 c | 176±20 g | 5687±514 a | 51.8 |
| J3 | 84.7±11.3 ab | 258.3±13.6 ab | 2783±141 cd | 164±22 g | 5610±341 a | 48.1 |
| J4 | 53.0±13.0 b | 167.7±5.5 bc | 2320±103 de | 162±16 g | 6053±69 a | 36.6 |
| J5 | 85.3±11.6 ab | 181.7±65.1 abc | 1573±382 f | 137±10 g | 4713±269 b | 31.4 |
| LSD(p<0.05) | 33.7 | 102.5 | 687 | | |  |
| **Wild-soybean accessions from Russia** | | | | | | |
| R1 | 47.3±8.7 ab | 150.3±28.9 ab | 1640±193 c | 73±7 d | 4953±487 a | 32.1 |
| R2 | 25.3±5.4 bc | 153.7±45.7 ab | 2868±833 b | 95±7 d | 5530±725 a | 51.0 |
| R3 | 22.7±1.5 bc | 37.7±3.2 c | 2873±554 b | 118±7 d | 2967±276 b | 96.7 |
| R4 | 17.3±0.3 c | 70.7±8.4 bc | 1419±189 c | 124±9 d | 3457±224 b | 38.9 |
| R5 | 62.3±14.9 a | 160.3±27.9 a | 2790±575 b | 126±13 d | 4960±572 a | 55.1 |
| LSD(p<0.05) | 25.6 | 86.7 | 1064 | | |  |
| **Wild- soybean accessions from China** | | | | | | |
| CH1 | 118.0±9.3 a | 313.0±14.0 a | 2707±197 c | 105±13 e | 4490±487 ab | 59.3 |
| CH2 | 105.7±11.3 ab | 265.3±24.0 ab | 2570±438 c | 93±8 e | 4227±166 b | 59.9 |
| CH3 | 61.7±5.8 bc | 33.7±42.2 abc | 2750±483 c | 129±26 e | 4890±92 a | 55.1 |
| CH4 | 52.7±2.2 c | 149.0±7.6 c | 1190±36 d | 83±9 e | 3577±160 c | 31.7 |
| CH5 | 92.0±31.4 abc | 198.0±55.9 bc | 2533±490 c | 240±16 e | 4813±372 a | 50.1 |
| LSD(p<0.05) | 49.6 | 106.7 | 703 | | | |

Numbers refer to mean values (±SE, n=3) of three Leonard jars, each containing two plants. Determinations were carried out 60 days after inoculation. For each parameter and accessions of the same country, data followed by the same letter are not significantly different at α = 5 %. n.s. = non-significant.

The Relative Efficiency Index (REI) for each inoculant/soybean-accession combination was calculated by comparisons among Plant-Top Dry-Weight (PTDW) of inoculated treatments (I), N-fertilized (N) and Untreated (U) controls. REI values were obtained by using the relation (I - U/ N- U) x 100.
