# Supplementary material for: Sinorhizobium fredii Strains HH103 and NGR234 Form Nitrogen Fixing Nodules With Diverse Wild Soybeans (Glycine soja) From Central China but Are Ineffective on Northern China Accessions
Source: Front Microbiol. 2018 Nov 21;9:2843. doi: 10.3389/fmicb.2018.02843 (PMC6258812; doi:10.3389/fmicb.2018.02843)
Supplement: Supplementary file 6 [file Table_6.docx]

**Table S6.** Symbiotic responses of *Glycine soja* accessions from North and Central China to the inoculation with *B. diazoefficiens* USDA110^T^.

| **Accession** | **Number of nodules** | | **Dry weight of nodules (mg)** | **Shoot dry-weight (mg) of plants** | | | **REI**  **(%)** |
| --- | --- | --- | --- | --- | --- | --- | --- |
|  |  |  |  | **Inoculated** | **Uninoculated** | **N-fertilized** |  |
| **Wild-soybean accessions from North of China (Heilongjiang and Jilin provinces)** | | | | | | | |
| CH6 | 106.7±44.5 abc | | 192.7±68.1 bc | 3085±331 def | 88±4 j | 3423±511 de | 89.9 |
| CH7 | 29.0±5.1 d | | 47.3±6.4 d | 1100±22 i | 69±15 j | 3187±156 def | 33.1 |
| CH8 | 41.0±16.2 cd | | 129.7±58.5 abcd | 1587±655 hi | 94±4 j | 4810±166 bc | 31.7 |
| CH9 | 84.3±40.1 abcd | | 86.0±18.2 cd | 2004±540 gh | 133±10 j | 3840±297 cd | 50.5 |
| CH10 | 82.3±8.4 abcd | | 218.7±21.2 bc | 1850±11 ghi | 130±12 j | 5873±420 a | 29.9 |
| CH11 | 127.0±2.0 ab | | 229.0±44.0 b | 1820±410 ghi | 87±2 j | 5660±390 ab | 31.1 |
| CH12 | 133.0±24.6 a | | 236.0±49.8 ab | 2653±708 efg | 70±13 j | 5430±176 ab | 48.2 |
| CH13 | 63.7±16.8 bcd | | 228.3±67.2 b | 1890±509 ghi | 90±6 j | 4850±166 b | 37.8 |
| CH14 | 54.7±6.5 cd | | 168.3±27.2 bcd | 1690±324 hi | 85±8 j | 5400±301 ab | 30.2 |
| CH15 | 139.0±24.7 a | | 366.7±46.8 a | 2327±367 fgh | 116±6 j | 4697±363 bc | 48.3 |
| LSD  (p<0.05) | 33.1 | | 134.3 | 900 | | |  |
| **Wild-soybean accessions from Central China (Shaanxi and Shanxi provinces)** | | | | | | | |
| CH16 | 78.7±18.1 bc | 185.7±36.1 | | 1857±323 ef | 118±22 h | 4023±832 cd | 44.5 |
| CH17 | 63.7±4.4 cd | 188.3±21.1 | | 1600±200 ef | 79±27 h | 4093±532 bcd | 37.9 |
| CH18 | 120.7±28.1 a | 223.3±20.0 | | 1933±104 e | 130±14 h | 5203±287 a | 35.5 |
| CH19 | 89.0±8.7 abc | 190.0±14.2 | | 1413±174 ef | 124±28 h | 4473±294 abc | 29.6 |
| CH20 | 60.3±10.3 cd | 196.3±2.0 | | 1613±67 ef | 122±9 h | 3343±64 d | 46.3 |
| CH21 | 52.3±14.1 cd | 122.0±38.0 | | 577±48 gh | 108±6 h | 4003±226 cd | 12.0 |
| CH22 | 29.5±9.5 d | 114.5±41.5 | | 1143±677 fg | 63±17 h | 4620±23 abc | 23.7 |
| CH23 | 63.7±18.4 cd | 148.0±41.5 | | 1726±544 ef | 102±13 h | 4027±190 cd | 41.4 |
| CH24 | 108.3±10.7 ab | 194.7±19.1 | | 1727±163 ef | 150±19 h | 4810±636 ab | 33.8 |
| CH25 | 76.7±4.1 bc | 185.7±19.8 | | 1690±179 ef | 146±18 h | 3940±276 cd | 40.7 |
| LSD  (p<0.05) | 41.7 | n.s. | | 748 | | |  |

Numbers refer to mean values (±SE, n=3) of three Leonard jars, each containing two plants. Determinations were carried out 60 days after inoculation. For each parameter and accessions of the same country, data followed by the same letter are not significantly different at α = 5 %. n.s. = non-significant.

The Relative Efficiency Index (REI) for each inoculant/soybean-accession combination was calculated by comparisons among Plant-Top Dry-Weight (PTDW) of inoculated treatments (I), N-fertilized (N) and Untreated (U) controls. REI values were obtained by using the relation (I - U/ N- U) x 100.
