# Supplementary material for: Sinorhizobium fredii Strains HH103 and NGR234 Form Nitrogen Fixing Nodules With Diverse Wild Soybeans (Glycine soja) From Central China but Are Ineffective on Northern China Accessions
Source: Front Microbiol. 2018 Nov 21;9:2843. doi: 10.3389/fmicb.2018.02843 (PMC6258812; doi:10.3389/fmicb.2018.02843)
Supplement: Supplementary file 7 [file Table_7.docx]

**Table S7.** Symbiotic responses of *Glycine soja* accessions from North and Central China to the inoculation with *B. elkanii* USDA76^T^.

| **Accession** | **Number of nodules** | **Dry weight of nodules (mg)** | | **Shoot dry-weight (mg) of plants** | | | | **REI (%)** |
| --- | --- | --- | --- | --- | --- | --- | --- | --- |
|  |  |  |  | **Inoculated** | | **Uninoculated** | **N-fertilized** |  |
| **Wild-soybean accessions from Northern China (Heilongjiang and Jilin provinces)** | | | | | | | | |
| CH6 | 32.3±3.0 d | | 65.3±9.2 de | | 727±134 g | 88±4 i | 3423±511 de | 19.2 |
| CH7 | 33.3±2.2 d | | 35.7±4.7 e | | 503±26 ghi | 69±15 i | 3187±156 e | 13.9 |
| CH8 | 67.3±1.2 bcd | | 93.0±19.3 cd | | 962±179 g | 94±4 i | 4810±166 c | 18.4 |
| CH9 | 32.0±5.6 d | | 60.0±16.8 de | | 818±95 g | 133±10 hi | 3840±297 d | 18.5 |
| CH10 | 88.3±12.5 bc | | 148.7±4.7 b | | 1673±52 f | 130±12 hi | 5873±420 a | 26.9 |
| CH11 | 148.7±23.2 a | | 206.3±33.8 a | | 1977±313 f | 87±2 i | 5660±176 a | 33.9 |
| CH12 | 105.7±13.3 b | | 123.0±22.1 bc | | 938±84 g | 70±13 i | 5430±176 a | 16.2 |
| CH13 | 79.0±21.8 bc | | 83.0±3.8 cde | | 688±134 gh | 90±6 i | 4850±166 bc | 12.6 |
| CH14 | 49.3±6.4 cd | | 95.3±1.2 cd | | 537±12 ghi | 85±8 i | 5400±301 ab | 8.5 |
| CH15 | 98.7±25.4 b | | 76.0±20.3 cd | | 516±159 ghi | 116±6 hi | 4697±363 c | 8.7 |
| LSD  (p<0.05) | 20.4 | | 49.7 | | 575 | | |  |
| **Wild-soybean accessions from Central China (Shaanxi and Shanxi provinces)** | | | | | | | | |
| CH16 | 73.7±21.9 bcd | | 125.7±48.3 bc | | 1750±580 e | 118±22 fg | 4023±832 cd | 41.8 |
| CH17 | 44.3±14.2 cd | | 66.0±30.7 cd | | 782±160 fg | 79±27 g | 4093±832 bcd | 17.5 |
| CH18 | 112.3±12.1 a | | 231.7±8.9 a | | 2437±267 e | 130±14 fg | 5203±287 a | 45.5 |
| CH19 | 91.0±11.8 ab | | 184.7±31.0 ab | | 2137±264 e | 124±28 fg | 4473±294 abcd | 46.3 |
| CH20 | 37.7±5.0 d | | 55.3±18.3 cd | | 673±98 fg | 122±9 fg | 3343±64 d | 17.1 |
| CH21 | 55.7±8.7 bc | | 45.3±0.3 d | | 548±34 fg | 108±6 fg | 4003±226 cd | 11.3 |
| CH22 | 43.7±4.7 c | | 46.7±1.3 d | | 555±45 fg | 63±17 g | 4620±23 abc | 10.8 |
| CH23 | 61.0±13.1 b | | 76.3±12.3 cd | | 886±4 f | 102±13 g | 4027±190 cd | 20.0 |
| CH24 | 77.0±2.6 abc | | 153.0±4.0 b | | 1893±396 e | 150±19 fg | 4810±636 ab | 37.4 |
| CH25 | 76.0±5.6 abc | | 180.7±29.8 a | | 1849±531 e | 146±18 fg | 3940±276 cd | 44.9 |
| LSD  (p<0.05) | 37.4 | | 72.4 | | 781 | | |  |

Numbers refer to mean values (±SE, n=3) of three Leonard jars, each containing two plants. Determinations were carried out 60 days after inoculation. For each parameter and accessions of the same country, data followed by the same letter are not significantly different at α = 5 %. n.s. = non-significant.

The Relative Efficiency Index (REI) for each inoculant/soybean-accession combination was calculated by comparisons among Plant-Top Dry-Weight (PTDW) of inoculated treatments (I), N-fertilized (N) and Untreated (U) controls. REI values were obtained by using the relation (I - U/ N- U) x 100.
